# Impact of a Pulse-Enriched Human Cuisine on Functional Attributes of the Gut Microbiome Using a Preclinical Model of Dietary-Induced Chronic Diseases

**DOI:** 10.3390/nu16183178

**Published:** 2024-09-20

**Authors:** Tymofiy Lutsiv, Elizabeth S. Neil, John N. McGinley, Chelsea Didinger, Vanessa K. Fitzgerald, Tiffany L. Weir, Hisham Hussan, Terryl J. Hartman, Henry J. Thompson

**Affiliations:** 1 Cancer Prevention Laboratory, Colorado State University, Fort Collins, CO 80523, USA; tymofiy.lutsiv@colostate.edu (T.L.); elizabeth.neil@colostate.edu (E.S.N.); vanessa.fitzgerald@colostate.edu (V.K.F.); 2 Graduate Program in Cell and Molecular Biology, Colorado State University, Fort Collins, CO 80523, USA; tiffany.weir@colostate.edu; 3 Department of Food Science and Human Nutrition, Colorado State University, Fort Collins, CO 80523, USA; chelsea.didinger@colostate.edu; 4 Department of Internal Medicine, University of California, Davis, Sacramento, CA 95817, USA; hhussan@ucdavis.edu; 5 Department of Epidemiology, Rollins School of Public Health, Emory University, Atlanta, GA 30322, USA; terryl.johnson.hartman@emory.edu

**Keywords:** beans, chronic disease, cuisine, dietary pattern, gut microbiome, microbial ecosystem, pulses

## Abstract

Introducing grain legumes, i.e., pulses, into any food pattern effectively increases dietary fiber and other bioactive food components of public health concern; however, the impact depends on the amount consumed. Given the convergence of preclinical and clinical data indicating that intake of at least 300 g (1.5 cup) of cooked pulse per day has clinically observable benefit, the feasibility for a typical consumer was demonstrated by creation of a fourteen-day menu plan that met this criterion. This menu plan, named Bean Cuisine, was comprised of a combination of five cooked pulses: dry beans, chickpeas, cowpeas, dry peas, and lentils. As reported herein, the impact of each menu day of the fourteen-day plan on gut microbial composition and predicted function was evaluated in female C57BL/6J mice, a strain commonly used in studies of metabolic dysfunction-associated chronic diseases. We report that pulse-related effects were observed across a wide variety of food item combinations. In comparison to a pulse-free human cuisine, all pulse menu days enriched for a gut ecosystem were associated with changes in predicted metabolic pathways involving amino acids (lysine, tryptophan, cysteine), short-chain fatty acids (butyrate, acetate), and vitamins (B_1_, B_6_, B_9_, B_12_, K_2_) albeit via different combinations of microbiota, according to the PICRUSt2 estimates. The predicted metabolic functions correlating with the various pulses in the menus, indicate the value of a food pattern comprised of all pulse types consumed on a regular basis. This type of multi-pulse food pattern has the potential to enhance the taxonomic and functional diversity of the gut microbiome as a means of strengthening the resilience of the gut ecosystem to the challenges associated with the daily activities of living.

## 1. Introduction

According to estimates from the World Health Organization, seven of the top ten leading causes of mortality worldwide in 2019 were noncommunicable diseases (NCD, also called chronic diseases), accounting for 74% of all deaths [1]. The rate of deaths associated with chronic diseases has increased by 75% since 2000 and is projected to account for 86% of all deaths worldwide by 2048 [2]. The development of NCD is based on genetic, physiological, environmental, and behavioral factors and their combinations. Leading global risks to health overall, especially in the context of NCD burden, are low dietary quality and physical inactivity. Therefore, while genetic predisposition and socioeconomic environment cannot be easily changed, alterations in dietary patterns can represent a more feasible approach to elicit a meaningful impact on the onset of NCD. 

Modern lifestyles increase the likelihood that people will eat in excess, consume foods high in sodium, added sugars, and saturated fat, and combine the intake of high-energy foods with reduced energy expenditure through physical activity [3]. This lifestyle frequently leads to metabolic dysregulation, resulting in hypertension, hyperglycemia, hyperlipidemia, being overweight or obese, and other critical metabolic risk factors underlying the development of NCD [4]. The World Health Organization considers a diet to be healthy if per day it provides less than 10% of total energy intake from added sugars, less than 30% of total energy from fats, and less than 5 g of salt, while containing legumes, whole grains, nuts, and at least 400 g of fruits and vegetables [5]. The inclusion of healthy, nutrient-dense foods like legumes, fruits, and vegetables is derived from their low energy density accompanied by their macronutrient diversity, dietary fiber, minerals, vitamins, and bioactive phytochemicals. 

The prevalence of NCD has been associated with gut dysbiosis that results in intestinal barrier damage, inflammation, and toxicity from harmful products of bacterial metabolism, which are effects associated with eating patterns low in dietary fiber [6,7]. An accepted approach to improve dietary patterns is to maximize the consumption of whole foods that are healthful, nutritious, and affordable. These foods should also be produced sustainably and feasibly introduced into existing dietary patterns. Considering their associated human and environmental benefits, pulses are ideal candidates for such foods, particularly considering the rich culinary history of pulses in cuisines around the world [8]. Due to their high dietary fiber content, including insoluble and soluble fiber and galacto-oligosaccharides in the raffinose family, pulses have a low glycemic index [9]. The increased satiety that can result after the intake of pulses combined with their nutrient-dense nature makes them potent functional foods. In fact, higher and regular levels of pulse consumption are associated with a reduced incidence of NCD. In preclinical studies, pulses have been found to induce dramatic changes in gut microbial composition, including a reduction in the abundance of bacteria associated with an unhealthy gut [10,11], supporting the role of the gut microbiota in mediating the beneficial effects of pulses. 

Given the convergence of preclinical and clinical data indicating that intake of at least 300 g (1.5 cup) of cooked pulse per day has clinically observable benefit [12,13,14], feasibility for a typical consumer was demonstrated by the creation of a fourteen-day menu plan that met this criterion [15]. This menu plan, which we refer to as the Bean Cuisine, was comprised of a combination of five cooked pulses: dry beans, chickpeas, cowpeas, dry peas, and lentils. The experiment reported here was designed to determine how each day’s menu of Bean Cuisine would affect gut microbial communities and their predicted function in a widely recognized model for dietary-induced obesity in female C57BL/6J mice in which effects of pulses on gut microbiome composition and function have been reported, although in male animals [16,17]. 

## 2. Materials and Methods

### 2.1. Study Design

Bean Cuisine is a 2-week menu plan that provides a daily dose of at least 300 g of cooked pulse, providing an average of 39.8% of total dietary protein per day. Based on analysis using Nutritionist Pro, version 8.1.0 (Axxya Systems, LLC, Redmond, WA, USA), Bean Cuisine is estimated to provide the recommended 1999 kilocalories per day from 292 g of carbohydrate, 59 g of fat, and 96 g of protein. Therefore, Bean Cuisine meets dietary recommendations, especially in the daily intake of dietary fiber (61 g) and potassium (5143 mg), which are typically under-consumed in the U.S. [18]. More detailed data are provided (Supplementary Materials S1 and S2). 

The recipes and respective side dishes that comprised each day of the Bean Cuisine menu (breakfast, lunch, snack, and dinner) were combined into a single homogenized experimental diet per each menu day; resulting in fourteen menus labeled by their respective day, D01–D14 with each menu day representing an experimental group. Each experimental group will be referred to as “menu” herein. e.g., D01 is group 1, D02 is group 2, through D14, etc. Colorado State University Housing and Dining Services (CSU HDS) assisted in the formulation and preparation of a comparable pulse-free menu plan which is referred to as the control menu, which is representative of what is served in the dining halls. The nutritional profile for the control menu is presented (Supplementary Materials S3). Proximate analysis was performed on each of the menu days and control diet (Table 1).

**Figure 1:**
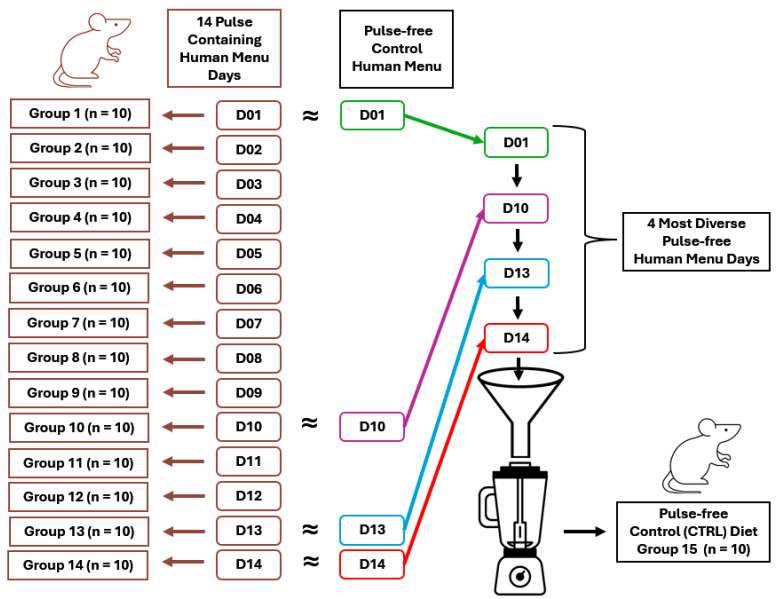
Study design. Animals were housed *n* = 10/group, 2 cages, 5/cage.

C57BL/6J mice (cat#000664, *n* = 150), known as the classic animal model of diet-induced obesity, were selected for this study. Female mice were used for three reasons: (1) while the microbiome of both male and female mice respond to dietary pulse, the magnitude of the response is greater in female mice, making them a useful choice for an initial study that screened all menu days of the Bean Cuisine; (2) female mice are underrepresented in pulse-microbiome research [11]; (3) the ratio of gut bacteria to host cells in females is greater than in males (2.2 vs. 1.3, respectively) [19], inferring a greater potential impact of the gut microbiome on female health. Mice were received at 7 weeks of age from the Jackson Laboratory (Barr Harbor, ME, USA) and maintained via standard husbandry conditions. Mice were housed in solid-bottom polycarbonate mouse cages and had ad libitum access to filtered water and food. They were exposed to a 12 h light/dark cycle and 27.5 ± 2 °C ambient temperature. Animals were adapted to the husbandry routine and a purified powdered diet for two weeks (based on formulation D12266B by Research Diets Inc., New Brunswick, NJ, USA) to provide a moderate amount of fat-derived energy (32% kcal). After acclimation, ten mice were assigned via stratified randomization by body weight to each experimental group (fourteen pulse-containing menu groups (D01–D14) and one pulse-free control (CTRL) group) and fed their respective Bean Cuisine menu for nine weeks as shown in the study design (Figure 1). Additional details on housing conditions and animal care were reported in [11]. All animal-related work was performed in accordance with the Colorado State University Institutional Animal Care and Use Committee of Colorado State University (Protocol KP#5432). 

### 2.2. Microbiome Analysis

Microbial DNA was isolated from cecal content, sequenced, and microbial composition analyzed based on the sequencing of data from the 16S rRNA gene’s V4 region as previously described [11]. Samples with less than 10,000 sequenced reads (*n* = 2, one from group D02 and one from group D14) were excluded from the subsequent analysis (Supplementary Table S8). The data were processed in QIIME 2 (version 2023.2) [20]. The Greengenes2 reference database was used to map amplicon sequence variants (ASV) to bacterial taxonomy [21]. Predictions of microbial functions were performed using the PICRUSt2 (Phylogenetic Investigation of Communities by Reconstruction of Unobserved States) pipeline [22]. Briefly, MetaCyc and KEGG pathway abundances were calculated from E.C. numbers and KEGG IDs of the genes whose frequencies were predicted based on the taxonomic assignments applied to the ASV dataset. 

### 2.3. Effect Size and Power

The primary outcome variable for this study on which sample size was computed was β-diversity of microbiota [23]. Weighted UniFrac distances were used to compute effect size, sample size, and power from our common bean dose-response study [11]. For all dietary doses of common bean, the effect sizes ranged from 0.497 to 2.664, as indicated in Supplementary Table S9. The effect size for the bean dose equivalent to this study (35% of total dietary protein from common bean) was 1.409. For the smallest effect size, 80% power was achieved for alpha = 0.05 with 8 mice per group. For the bean dose of 35% total protein equivalent to this study, the sample size was 3 mice per group. Therefore, the sample size for this experiment was set to 10 mice per group, exceeding the power calculated even for observed effects with the lower dose of common bean (Supplementary Table S9). 

### 2.4. Statistical Analyses

α- and β-diversity of microbiota isolated from cecal content were analyzed in QIIME 2 using observed characteristics, Faith’s phylogenetic diversity, Pielou’s evenness, Shannon’s diversity index (all statistically compared using the Kruskal–Wallis method), and Jaccard, Bray–Curtis, weighted and unweighted UniFrac distances (tested using permutational multivariate analysis of variance (PERMANOVA) and permutational analysis of multivariate dispersions (PERMDISP) methods), respectively. 

Differential abundance analyses were performed using several techniques: on the phylum level, the Kruskal–Wallis and its post hoc Dunn testing were performed on the total sum-scaled (TSS) relative abundance of bacteria in *R* (version 4.4.0). On the genus level, the linear discriminant analysis (LDA) effect size (LEfSe) method [24] in MicrobiomeAnalyst 2.0 [25] and ANCOM with bias correction (ANCOM-BC [26]) method in QIIME 2 were conducted. Differential abundance analysis of the PICRUSt2-derived functional predictions was performed using the ANCOM-BC method in QIIME 2 to infer statistically significant differences between the groups.

Spearman correlation analysis between the food components, bacterial abundance, and their functional predictions was performed in JMP Pro (version 16.2.0). 

## 3. Results

During the study, there were no observable adverse effects to any of the diet formulations fed to animals. Of the 150 mice assigned to the experiment, evaluable data were obtained from 139 animals (92.6%). Of the eleven mice not evaluated, three did not survive to the end of the study; one mouse was discovered to have only one kidney at necropsy; one mouse had a large kidney cyst; one mouse had chronic malocclusion. Additionally, three mice had body weights that were statistical outliers, and data analyses were done with these animals included or excluded, revealing no effect on outcomes. The data presented were with these animals excluded since this improved the fit of statistical models. Finally, two samples had low reads (<10,000) of the 16S rRNA gene amplicon data that resulted in a rejection based on quality control criteria. This information is summarized in Supplementary Table S8.

### 3.1. Bean Cuisine Menus Increase Diversity of Cecal Microbiota

**Figure 2:**
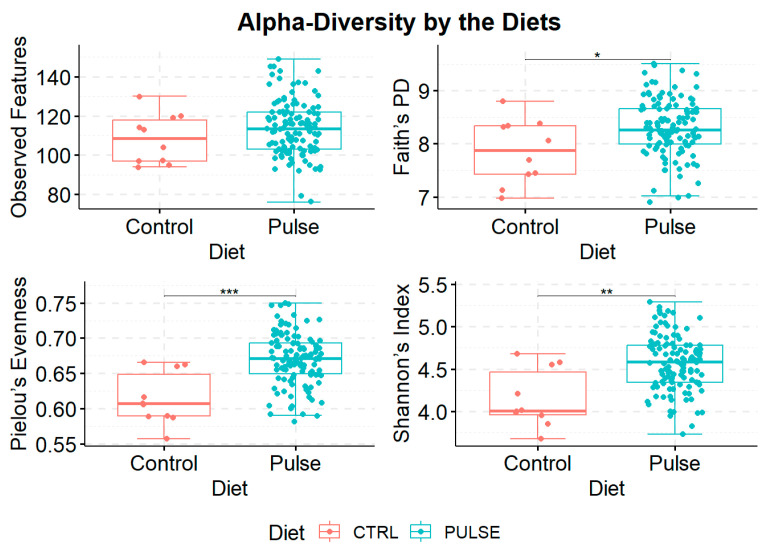
Measurements of α-diversity of the cecal microbiota of mice fed pulse-free control and pulse-containing menus combined. Statistical comparisons between the groups were conducted using the Kruskal–Wallis test where * *q*-value < 0.05, ** *q*-value < 0.01, and *** *q*-value < 0.001.

To determine the community-wide effects of pulse cuisine menus, we investigated how diverse microbial composition was within samples (α-diversity). Considering higher content of the dietary fiber in Bean Cuisine, we anticipated an increase in microbial α-diversity in the ceca of mice. Although the presence of ASVs per sample was similar across samples (Figure 2 top-left), other metrics of α-diversity indicated significant enrichment of microbiota in animals within the pulse-containing menus. Specifically, Pielou’s metric indicated that proportions between ASVs were more even in the pulse cohort (Figure 2, bottom-left), and according to Faith’s phylogenetic diversity metric, pulse enriched the ceca with a greater taxonomical variety of microbiota (Figure 2, top-right). Finally, Shannon’s index combines information about the richness and abundance of unique ASVs and showed an increased diversity thereof in the pulse-containing menus compared to the control (Figure 2, bottom-right). 

**Figure 3:**
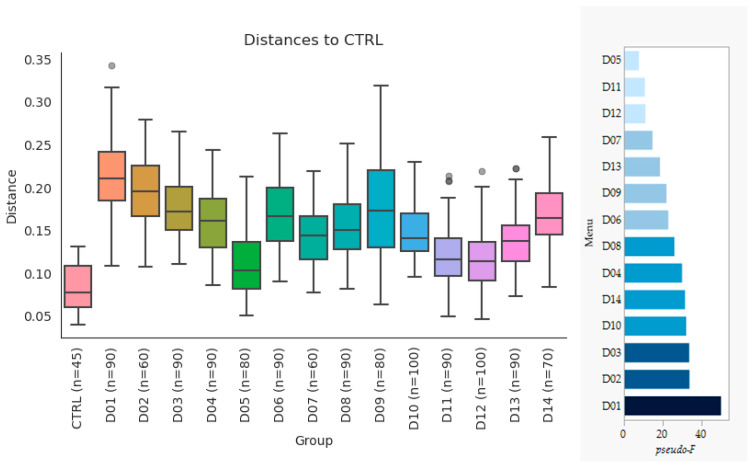
Weighted UniFrac distances of the β-diversity across Bean Cuisine menus and pulse-free control. Group PERMANOVA results: pseudo-*F* = 12.81, *p*-value = 0.001. *n* represents amount of ASVs included in diversity analysis after rarefaction. The pairwise PERMANOVA pseudo-*F* values for each menu vs. control are ranked on the bar graph to the right (all *q*-values < 0.01).

The impact on the cecal microbial composition of the fourteen unique menus of Bean Cuisine were contrasted amongst one another and with the pulse-free control. β-diversity analysis revealed that all pulse menu days were statistically distinct from the control according to the Jaccard, Bray–Curtis, unweighted and weighted UniFrac indices (Supplementary Table S1). The weighted UniFrac, which combines information on phylogenetic diversity with the abundance of bacteria, most clearly represented the difference between Bean Cuisine and the pulse-free control (Figure 3). Menus D01–D03 induced the most distinct microbial communities from the pulse-free control. The menus D05, D11, and D12 differed the least from the control. 

### 3.2. Individual Bean Cuisine Menus Induce Distinct Microbial Signatures

**Figure 4:**
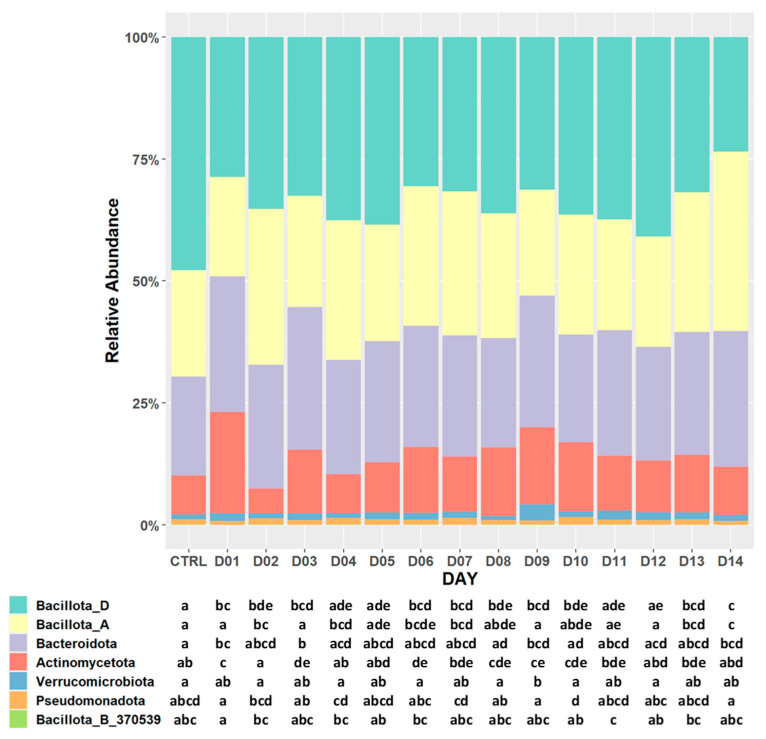
Bar plot of relative abundances of observed bacterial differences at the phylum level in pulse-containing and pulse-free control diets. The letters in the plot represent pairwise statistical comparisons using the Dunn test: matching letters within a particular row (phylum) among different columns (menu) denote the lack of statistical significance between the respective diet groups (*q*-value > 0.05).

Differences in the composition of bacterial communities across the experimental diet groups are best visualized through the relative abundances of their phyla (Figure 4). Bacillota_D were the most abundant in the pulse-free control group (47.48%) and the least present in D14 (23.51%). D14 had the highest relative abundance of Bacillota_A (36.76%) among other menu groups. D01 exhibited the lowest percentage of Bacillota_A (20.55%) and the absence of Bacillota_B_370539 clade, which was overall the least represented phylum in all menu days (<0.1%). Bacteroidota were the least abundant in the ceca of the control relative to other menus, and their increase was the most significant in D01, D03, D09, and D14. 

**Figure 5:**
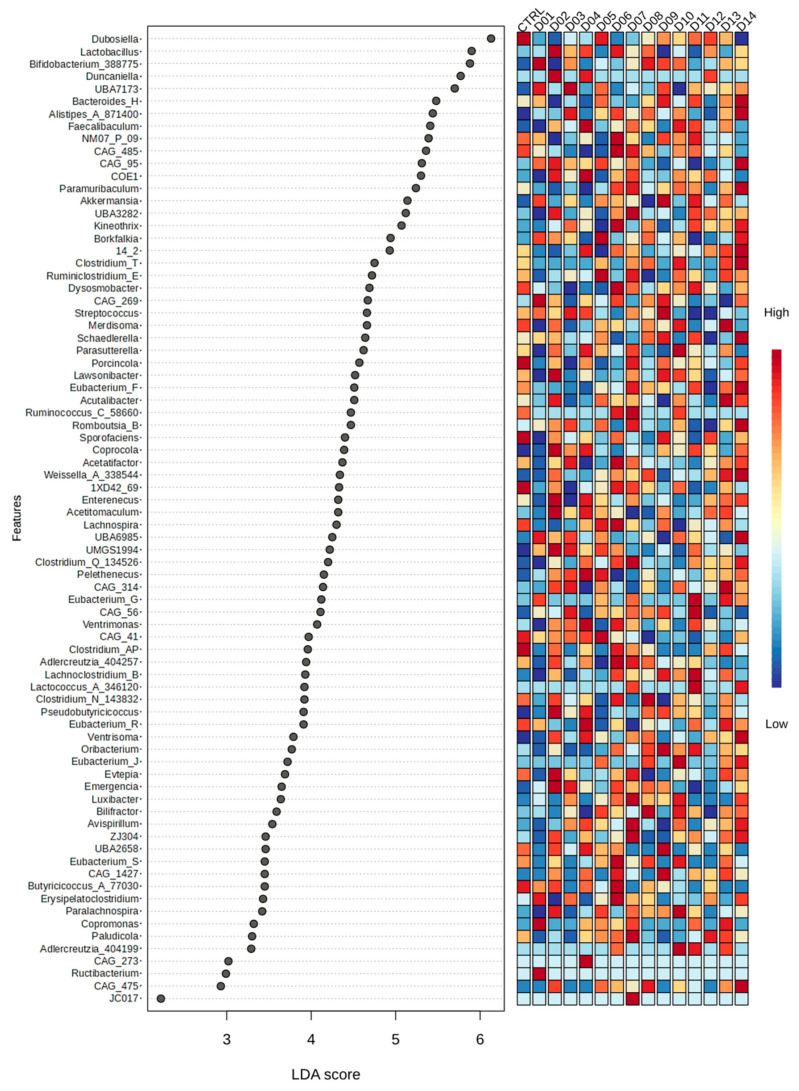
Differential abundance analyses of microbial differences across pulse-free control and fourteen pulse-based menus from Bean Cuisine using LEfSe with cutoffs for |LDA score| > 2 and *q*-value < 0.05. The full report can be found in Supplementary Table S2.

To understand differences in the bacterial composition on the deeper level of taxonomic organization, we performed differential abundance analysis using LEfSe and ANCOM-BC methods. Seventy-eight bacterial genera were most likely to explain the differences between the menus (Figure 5, Supplementary Table S2). Compared to the pulse-free control, Bean Cuisine suppressed the abundance of *Dubosiella* sp., *CAF-41*, *CAG-485*, *Clostridium_T* sp., *Lachnospira* sp., *Merdisoma* sp., *Porcincola* sp., *Streptococcus* sp., and *1XD42-69* sp. statistically in at least half of the menus according to ANCOM-BC (Supplementary Table S3). The presence of more bacterial taxa was enhanced, instead: *Alistipes_A_871400* sp., *Bifidobacterium_388775* sp., *Coprocola* sp., *Pelethenecus* sp., *Pseudobutyricicoccus* sp., *UBA7173* sp., *UMGS1994* sp., and *Ventrisoma* sp. increased in seven or more menus. Among them, *Coprocola* sp. was enhanced in all fourteen menus of Bean Cuisine compared to the pulse-free control. *Streptococcus* sp. was suppressed in nine yet was enhanced in two of Bean Cuisine menus compared to the control (Supplementary Table S3). 

### 3.3. Functional Predictions of the Bean-Culinary-Driven Microbiota

**Figure 6:**
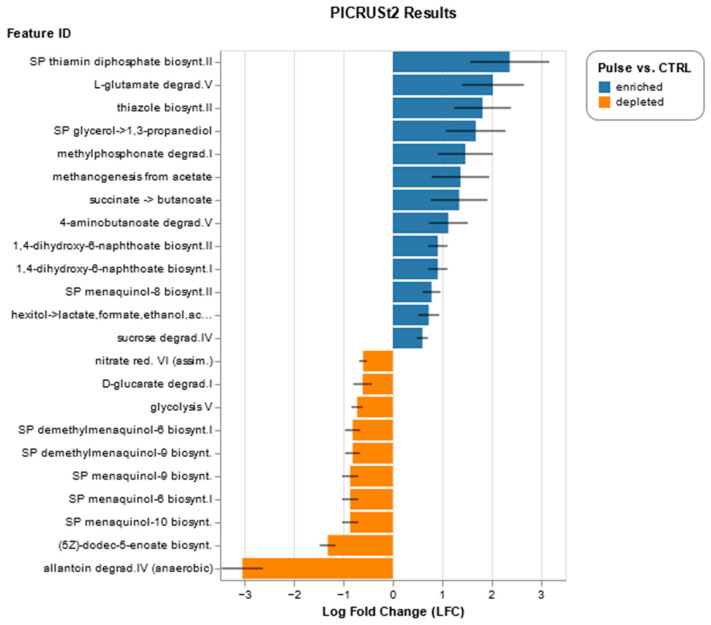
Functional predictions of microbial functions in Bean Cuisine vs. pulse-free control: ANCOM-BC results on combined pulse-containing vs. pulse-free groups (|log_2_ fold change| > 0.6; *q* < 0.05). Full report can be found in Supplementary Table S4.

Predictions about the functional capabilities of microbial signatures based on their taxonomical composition offer insightful information about metabolic processes potentially dominated by different menus of Bean Cuisine. Using Phylogenetic Investigation of Communities by Reconstruction of Unobserved States version 2 (PICRUSt2), we applied multiple approaches to focus on the consistent metabolic capacity of microbiota in the ceca of mice. Overall, more metabolic pathways were predicted to be enhanced in the Bean Cuisine versus control than suppressed. Production of butyric acid—an important short-chain fatty acid (SCFA) for gut health—was enhanced compared to control (Figure 6, Supplementary Table S4). Succinate fermentation to butyric acid was predicted to be predominantly driven by the increased bacteria in D01, D07, D08, D10, and D14. In contrast, the rest of the menus did not reach statistical significance despite large log_2_ fold change values. Pyruvate fermentation to butyric acid was enhanced by D02- and D13-driven bacteria. The D02 menu additionally enriched bacteria predicted also production of acetate and butyric acid from lysine fermentation. Another potential source of butyric acid production was enhanced by the bacteria in half of the Bean Cuisine menus owing to 4-aminobutyric acid (GABA) degradation with the pathway log_2_ fold change > 1.2, as well as glutamate degradation via hydroxyglutarate (log_2_ fold change > 2.5). Indeed, the predicted capacity for the GABA shunt was upregulated, involving *gadA/B/D/T* and *puuE* (Supplementary Table S5). All but D02, D04, D07, and D12 menus reached statistical significance with a slight increase in pyruvate fermentation to propanoate—another gut microbiota-synthesized SCFA with health benefits to the host. In contrast, pyruvate fermentation to acetate and lactate pathways was estimated to be reduced by D01, D07, D09, D13, and D14 (FDR-corrected *p*-value < 0.05). Therefore, the Bean Cuisine menus seem to induce bacteria predicted to possess a higher capacity of SCFA production. 

Bean Cuisine menus also increased bacteria predicted to ferment hexitol to lactate, formate, ethanol, and acetate. Moreover, the pathway for methanogenesis from acetate was also enriched in the Bean Cuisine cohort (Figure 6, Supplementary Table S6), predicting an increased coenzyme M output which, in turn, enables bacterial metabolism of toxic alkenes to anti-inflammatory acetoacetate. The allantoin degradation pathway in the gut bacteria, which is reported to be associated with obesity, was strongly reduced.

Consistent evidence was observed for Bean Cuisine-driven cecal microbiota predicted upregulation of vitamin and cofactor production. The menus are predicted to enrich bacterial capacity to produce thiamine (vitamin B_1_) compared to the control (Supplementary Table S4). Multiple menus of Bean Cuisine showed a higher presence of microbial *thiL*, *thiDE*, *thiF*, and other genes involved in the production of mono- and pyrophosphate. PICRUSt2 estimated a higher biosynthetic abundance of *mgnA–E* (vitamin K_2_/menaquinone), *ubiA/D/X* (coenzyme Q10/ubiquinone), *cobK/cbiJ*, *cbiK*, *MMAB/pduO* (vitamin B_12_/cobalamin), *pdxS*, *pdxT* (vitamin B_6_/pyridoxal-P), *sulD*, *folB*, *phoA/B* (tetrahydrofolate/vitamin B_9_ derivative) among other vitamin-like metabolites. In contrast, individual phylloquinone (*menI*) and vitamin B_2_/riboflavin (*RIB7/arfC*) biosynthetic genes were predicted to be downregulated instead (Table 2, Supplementary Table S6). Additionally, biosynthetic pathways of heme and lipoic acid were also predicted to be upregulated by the pulse-based Bean Cuisine. Therefore, consumption of the Bean Cuisine enhanced the potential for the gut microbiota to produce vitamins and their cofactors, which in turn increases the potential health benefits for the host. 

Bean Cuisine-driven bacteria also exhibited a higher capacity for sulfur metabolism, especially in reducing sulfate to H_2_S via upregulating both dissimilatory (*aprA/B*) and cysteine-synthesizing assimilatory pathways (*cysNC* and *cysP/U/W*) pathways’ enzymes. Cysteine biosynthesis is most likely to occur through the conversion of homocysteine (Table 3). Reconstruction of the amino acid metabolism varied with increased production of lysine, tryptophan, cysteine (from homocysteine), glutathione, and betaine across most Bean Cuisine menus. Synthesis of cysteine (not from homocysteine), histidine, phenylalanine, and tyrosine showed higher functional potential in the control diet (Table 4). Biosynthesis or degradation of sulfur-containing amino acids (cysteine, homocysteine, methionine, and taurine) were distinct between the control and the Bean Cuisine diets (Supplementary Tables S5 and S6). There is a consistent signature of amino acids produced and degraded in the Bean Cuisine diets vs. control.

### 3.4. Similarities and Differences in Microbial Responses to Bean Cuisine Menus

**Figure 7:**
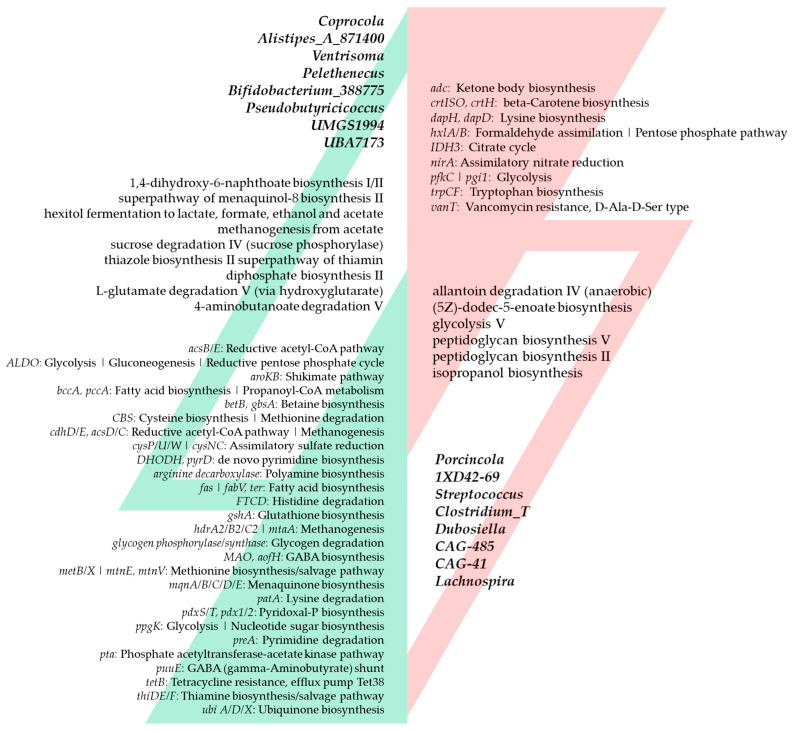
Bean Cuisine-induced ecogroups of the cecal bacteria and their predicted metabolic pathways. Features that are up-regulated in seven or more out of fourteen menus of Bean Cuisine are highlighted on the left (green), while on the right, respectively, are those that are suppressed in seven or more (red) out of fourteen menus of Bean Cuisine compared to the pulse-free control. Bacteria and pathways are organized within each list by the number of menus where they exhibit a significant change. Changes observed in at least half of Bean Cuisine menus compared to the pulse-free control were deemed significant with *p*-value < 0.05, *q*-value < 0.05, and |log_2_ fold change| > 0.6 according to the differential abundance analysis using the ANCOM-BC method.

Despite the diverse and rich formulation of Bean Cuisine, the common denominator that unites all fourteen menus is the pulses. We focused on common trends in microbial signatures driven by the Bean Cuisine. Figure 7 depicts bacteria and their predicted functions that were shared by the significant results of differential abundance analyses in at least half of the Bean Cuisine menus.

**Figure 8:**
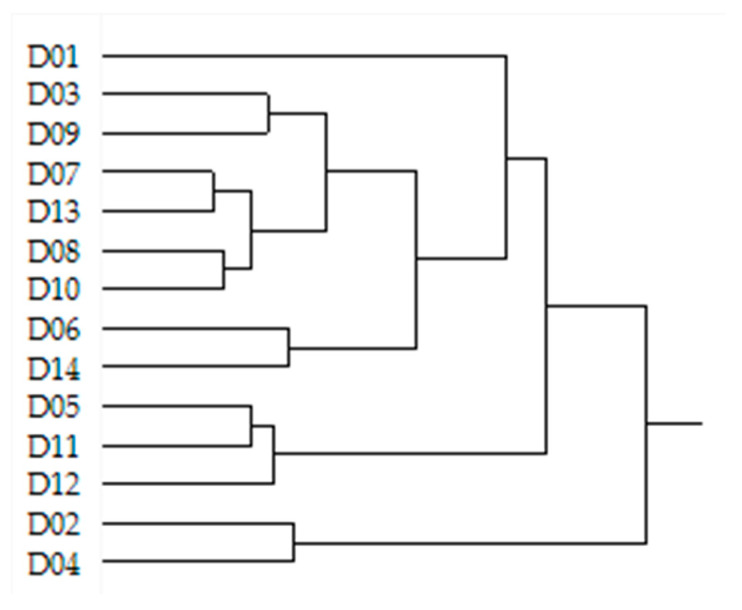
Clusters of Bean Cuisine menus based on the similar patterns of the cecal microbial composition they induce. Dendrogram represents the result of the hierarchical clustering analysis of the raw ASVs across fourteen menus of Bean Cuisine using the Ward method.

The microbial differences among the fourteen menus of Bean Cuisine from the pulse-free control show inequivalent patterns. Therefore, we subjected our 16S rRNA gene abundance dataset to hierarchical clustering analysis using the Ward method (Figure 8). The menus were grouped according to similarities and differences in their microbial composition. Supplementary Tables S2 and S3 contain the results of differential abundance analyses between menus, as previously mentioned. 

**Figure 9:**
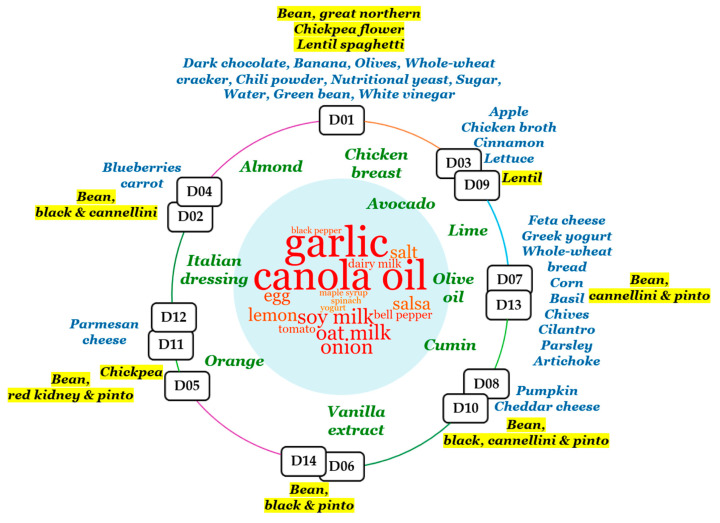
Distribution of food types across the microbiome-similar menus of Bean Cuisine. Clusters of menus are depicted on the circle. Inside the circle, the word cloud represents food types that were common across multiple clusters (from red, the most common, to green, shared by only two menu clusters). Outside of the circle, foods that were classifying each menu cluster are marked in blue and located near their cluster. Pulses present within each menu cluster are similarly located and highlighted in yellow. Clusters of menus were composed based on hierarchical analysis of the microbial composition that Bean Cuisine menus induce in the ceca of mice.

D01 was clearly distinct in microbial composition compared to all other Bean Cuisine menus. It contained the largest number of unique foods within its meals, such as dark chocolate, bananas, olives, whole wheat crackers, chili powder spice, and nutritional yeast. In addition to pinto and red kidney beans, D01 uniquely contained great northern bean, green bean (vegetable), lentil-based spaghetti, and chickpea flour (Figure 9). Compared to the control, D01 had a statistically increased abundance of *CAG-269* sp., *Copromonas* sp., and *Ructibacterium* sp., while *Lawsonibacter* sp. was uniquely suppressed in D01. As a result, pathways for D-galactarate degradation were significantly suppressed in D01-fed mice. 

Closest to D01 was the cluster of D03 and D09 menus. The latter uniquely contained apples, chicken broth, cinnamon spice, lettuce, and lentils (Figure 9). Furthermore, D01, D03, and D09 were the only menus that had an increase in *Streptococcus* sp. and a reduction in *Ruminiclostridium_E* sp. abundance. Furthermore, (5Z)-dodec-5-enoate biosynthesis and peptidoglycan biosynthesis II and V were enhanced in the ceca of these mice while being inhibited in the rest of the menus. Upregulated glucose and glucose-1-phosphate degradation, superpathway of sulfate assimilation and cysteine biosynthesis, mannan degradation, pyridoxal 5-phosphate biosynthesis I, as well as downregulated purine nucleotides degradation I (anaerobic) and II (aerobic) were prominent herein. Predicted increases in superpathways of demethylmenaquinol-8 and menaquinol-7/8/11/12/13 biosynthesis and in the abundance of *Akkermansia* were particularly observed in the D09 menu. 

One of the pulse types that appears in both menus D11 and D12 was red kidney bean, and both uniquely contained parmesan cheese. Dates, broccoli, and ketchup were only present in the D05 and D11 menus, where chickpea and pinto bean were the pulse source (Figure 9). These three menus (D05, D11, D12) were clustered together based on their microbial composition. Out of all the Bean Cuisine menus, the D05-D11-D12 cluster microbiota were the least distinct from the control and were not predicted to suppress the following: urate biosynthesis/inosine 5-phosphate degradation, superpathways of L-tyrosine, L-phenylalanine, L-aspartate, L-asparagine biosynthesis, L-lysine biosynthesis II, L-glutamate and L-glutamine, purine ribonucleosides degradation, adenine and adenosine salvage III, formaldehyde assimilation II (RuMP Cycle), and nitrate reduction VI (assimilatory).

Menus D06 and D14, which contained black and pinto beans, soy milk, eggs, bell pepper, and tomato, significantly inhibited octane and formaldehyde oxidation, as well as upregulated pyrimidine deoxyribonucleoside salvage and myo-inositol degradation.

Bacteria in the menus D02 and D04 selectively inhibited pyridoxal 5-phosphate biosynthesis I, pyrimidine deoxyribonucleotides de novo biosynthesis and phosphorylation, chondroitin sulfate degradation I, superpathway of sulfur oxidation, degradation of myo-, chiro-, and scillo-inositol. Unlike other menus, these menus included black and cannellini beans, carrots, and blueberries (Figure 9). *Acetitomaculum*, *COE1*, *Duncaniella*, *Lactobacillus* were among the enhanced genera by these menus, while *Bacteroides_H*, *Paramuribaculum*, *Clostridium_T*, and *Dysosmobacter* were among the suppressed ones.

### 3.5. Differential Patterns within Bean Cuisine Menus

**Figure 10:**
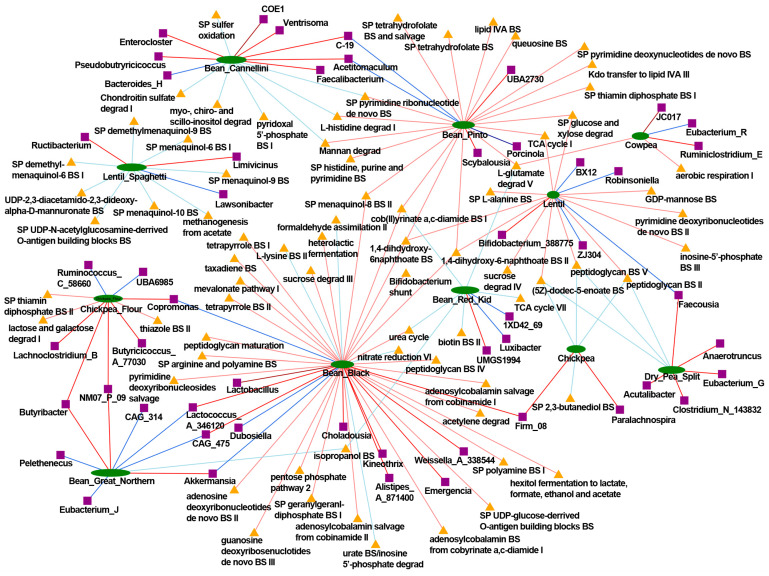
Network of pulse-associated cecal bacteria and their predicted pathways based on the correlation analysis. Pulse types are indicated in green, bacteria are indicated in purple, and pathways are indicated in yellow nodes. The respective nodes are connected by lines that indicate positive (red) or negative (blue) values of Spearman’s ρ. Here are only the relationships with |ρ| > 0.3 and *p*-value < 0.05. The full report can be found in Supplementary Table S7.

We further complemented our findings with the correlation analysis to infer associations among the bacteria, their functions, and the food components of Bean Cuisine menus. *Dubosiella* had the greatest effect size in the LEfSe results (LDA score = 5.77) and showed negative correlations with quinoa and black bean, but was positively associated with broccoli, garlic, and hot pepper sauce. Its abundance was suppressed compared with the control in all menus, except D05, D11, and D12. Most significantly, *Dubosiella* had positive correlation with L-lysine biosynthesis II and nitrate reduction VI, which, in turn, negatively correlated with black bean. *Streptococcus* was among the top differential bacteria, scoring highest by significance in LEfSe (*q*-value = 1.15 × 10^−18^; Supplementary Table S2). Pairwise analyses against the pulse-free control indicated that its abundance increased in the D01, D03, D04, and D09 menus (albeit with a relatively small effect size in D01 and D04) yet decreased in the rest of the Bean Cuisine menus. It scored a markedly high correlation with dairy yogurt (ρ = 0.937, *p*-value = 2.55 × 10^−7^; Figure 10, Supplementary Table S7), consistent with its amount in the D01, D03, D04, and D09 menus. It also achieved a high positive coefficient value with (5Z)-dodec-5-enoate biosynthesis, peptidoglycan biosynthesis II and V pathways (Supplementary Table S7). These pathways, in turn, were also positively associated with dairy yogurt and lentil, but negatively associated with chickpea and split pea in the diets. Abundance of *Streptococcus* was statistically indifferent only in D07. *Porcincola* was significantly suppressed in all menu clusters, and Spearman’s correlation analysis revealed that black bean had the most negative coefficient with this genus (ρ = −0.735, *p*-value = 1.79 × 10^−3^; Figure 10, Supplementary Table S7).

Pulse increased abundance of *Borkfalkia*—a non-dominant bacterial genus in humans whose species have been reported to promote health benefits after antibiotic exposure recovery as probiotics, most likely producing lactate, propionate, and acetate from various mono- and oligosaccharides (L-arabinose, fructan, L-fucoside, galactoside, β-glucoside, maltose, mannose, mannosides, L-rhamnoside, sucrose, trehalose/cellobiose, and xylan) [27]. This bacterium correlated significantly with L-glutamate degradation V across Bean Cuisine menus that exhibited negative associations with red kidney bean, but positively correlated with cowpea and pinto bean. *Alistipes* was significantly enriched in the pulse cohort compared to the control. Moreover, it reached significant positive correlation with black bean. Furthermore, 1,4-dihydroxy-6-naphthoate biosynthesis I and II, L-histidine degradation I, superpathway of menaquinol-8 biosynthesis II, superpathway of tetrahydrofolate biosynthesis had a highly positive correlation with *Alistipes* as well as with lentil, black bean, and pinto bean. 

## 4. Discussion

Consumption of pulses has been associated with a reduction in risk for or severity of various chronic diseases, including but not limited to cardiovascular disorders, type 2 diabetes, obesity, and cancer, potentially due to the large amounts of dietary fiber within cooked pulses. A convergence of clinical and preclinical data indicates that daily consumption of approximately 300 g (approximately 1.5 cups) of cooked common bean is well-tolerated and provides meaningful effects on the colon microbiome, improving metabolism and reducing colorectal cancer risk [12,28,29]. This level of intake also corresponds to the upper range of intake observed in the U.S. and Canadian populations [12,14,30]. Despite this, consumption of pulses in the United States is low, and it is often presumed that the 300 g/day dose of cooked pulse is not achievable. Thus, the Bean Cuisine was developed to demonstrate such feasibility. 

Bean Cuisine illustrates the implementation of an affordable, high dietary quality fiber-rich food pattern that effectively eliminates the dietary fiber gap, which has been linked to chronic disease burden [31,32]. The results reported herein are, to our knowledge, the first effort to determine the overarching effects of a pulse-centric food pattern comprised of multiple types of pulses fed at a level comparable to human dietary interventions of common bean that have been reported to be beneficial. In addition to the physicochemical properties of dietary fiber regulating intestinal motility, absorption, and even appetite, consumption of indigestible complex carbohydrates plays a crucial role in establishing intestinal health by modulating the microbial community and maintaining its stability. Metabolites of these microorganisms exert systemic effects for the host associated with human health and overall well-being, such as short-chain fatty acids (SCFAs), vitamins, and amino acids. In NCD prevention, the greatest attention is paid to SCFAs produced by the intestinal microbiota from dietary carbohydrates and proteins—acetate, butyrate, and propionate. SCFAs exert direct effects on intestinal cell physiology and gut health, as well as indirect effects on total body metabolism, appetite, innate and adaptive immunity, and the pathogenesis of various NCDs [7,33,34]. All Bean Cuisine menus were associated with predicted microbial metabolic pathways involving vitamins, amino acids, and SCFAs, albeit via a mixture of microbiota and metabolic pathways as illustrated in Figure 10. A key point derived from the examination of Figure 9 and Figure 10 addresses a common dietary trend. The use of pulses tends to focus on only one pulse type, usually common bean. Given the overall effects of pulse consumption as described above, Figure 9 provides a strong indication that at a sufficient pulse dose, pulse-related effects can be obtained across a wide variety of food item combinations with a hint of the potential value of foods that have probiotic content, for example, yogurt and cheese. In addition, Figure 10, which was a relational database-driven alignment of microbiota and their predicted metabolic functions correlating with the amounts of pulses in the menus, strongly speaks to the value of a food pattern comprised of all pulse types consumed regularly. In so doing, the gut microbiome’s pulse-driven taxonomic and functional diversity can be fully expressed in achieving and maintaining eubiosis.

### 4.1. Bean Cuisine-Induced Microbial Signatures

Through the lens of the gut microbiome as “an organ” determining overall health, our results indicate that consumption of any Bean Cuisine menu day enhanced the diversity of microbial composition and function relative to a healthy (according to WHO guidelines) but pulse-free dietary pattern. We observed several significant effects induced by dietary pulses on the cecal microbiome of mice, including community-wide taxonomical and functional differences from the pulse-free control. α-diversity of microbial communities shows that pulse-containing diets collectively show higher numbers and more evenly distributed bacterial taxa. We also showed that different bacteria and their functional pathways were associated with different types of pulses. Collectively, the functional arsenal of the intestinal bacteria was enhanced by Bean Cuisine, ensuring greater capacity to metabolize dietary components, modulate inter-microbial relationships, and increase the probability of the host harvesting the health benefits from microbial activity. Although increased individual diversity of the microbiota does not necessarily mean better health outcomes [11,35], an increase in the number of different microbiota increases the functional repertoire of their community, as well as the probability of beneficial effects from their activity. 

Among the bacteria affected the most, representatives of the Lachnospiraceae family showed the largest cluster. This family has a high capacity to utilize dietary carbohydrates and produce SCFAs, but despite strong implications in metabolic diseases and immune function, its role remains controversial [36]. Within this family, *Sporofaciens* were shown to be reduced after supplementation of the fermented fucoidan and λ-carrageenan in rats with Alzheimer’s disease compared to the high-fat diet-fed control [37]. *Porcincola* species were shown to produce lipase, deconjugate primary bile acids, and use cellulose and starch as the only sources of carbohydrate [38]. A decrease in *Lachnospira* as reported herein was implicated in bile acid metabolism when an intervention with dietary fiber, including from pea, was studied in the context of obesity [39].

Outside of the Lachnospiraceae family, *Eubacterium G* was enhanced in the pulse cohort. Overall, this genus is associated with health benefits in humans as it mainly produces butyrate and propionate, improves cholesterol and bile metabolism, modulates immune function, and contributes to the prevention of cardiovascular diseases and type 2 diabetes and the promotion of gut and liver health, showing comparable health benefits with *Lactobacillus* and *Bifidobacterium* [40]. Their abundance, along with fecal butyrate, was reported to increase in a fiber-rich diet, although it decreased in diet types that are commonly used as weight-loss strategies, with high dietary protein and fat and low carbohydrate [41].

*Alistipes* belongs to the Rikenellaceae family, which we previously reported to be in the pulse-enhanced bacterial ecogroup [11]. Various *Alistipes* sp. decrease with the progression of liver fibrosis and cirrhosis in patients with atrial fibrillation and in mice with ulcerative colitis, and their abundance was positively correlated with the prevention of autism spectrum disorder and with responsiveness to cancer immunotherapy. However, there are contrasting reports implicating this genus of bacteria with hypertension, colorectal cancer, and depressive mental disorders [42].

### 4.2. Comparison with Pulse Effects in Purified Diet Formulations in Rodents and with a Human Intervention

Previously, we reported the comparative cecal bacterial signatures from the consumption of bean, chickpea, dry pea, and lentils [43]. In comparison with the Bean Cuisine, *Alistipes_A_871400*, *Coprocola*, *Pseudobutyricicoccus*, *UBA7173*, *UMGS1994*, as well as *Borkfalkia* were enhanced in both studies. *Merdisoma* and *Streptococcus* were in the common suppressed ecogroup. The Bean Cuisine results were also similar to the microbial responses to our previously reported dose-response study using common bean in female and male mice [11]. In both experiments, *1XD42_69*, *Clostridium_AP*, *Clostridium_T*, *Dubosiella*, *Merdisoma*, *Porcincola*, *Sporofaciens*, and *Streptococcus* were suppressed, whereas *Alistipes_A_871400*, *Borkfalkia*, *Clostridium_Q_134526*, *Coprocola*, *Pelethenecus*, *Pseudobutyricicoccus*, and *UBA7173* were enhanced. Thus, the effect of pulses on gut microbial composition shared commonalities irrespective of whether provided in a purified diet formulation or as a component of a food mixture intended for human consumption. 

Recently, a common bean intervention (one cup per day) was evaluated in obese surveillance patients with a history of colorectal neoplasia in the BE GONE trial [12]. Similar to our results, the authors report an increase in the α- and β-diversity, an increase in *Bifidobacterium*, and a decrease in *Streptococcus*, a positive shift towards pyruvate fermentation, and a negative shift in lysine biosynthesis. Interestingly, the authors reported a decrease in thiazole biosynthesis—a component of thiamine (B_1_)—whereas our results show that Bean Cuisine increases production, thereof. Overall, bean consumption in BE GONE was associated with prebiotic efficacy and gut-healthy microbial and metabolic changes with therapeutic potential to improve obesity and colorectal cancer prospects. 

### 4.3. Value of Preclinical Modeling in Pulse Research

As recently reviewed by us [44], a major limitation in the population and clinical studies on pulse consumption and NCD endpoints is that most studies are underpowered from a statistical perspective. Moreover, study quality is heterogeneous and probably reflects limited funds spread across many types of pulses. We argue that preclinical studies can be part of an overall strategy to build support for well-funded experiments in humans and to assist with identifying biomarkers and candidate mechanisms. Many foundational studies required to design effective human intervention trials are simply not feasible to conduct on human subjects. Thus, in a time of a worldwide rise in the burden of metabolic dysfunction associated with diseases with long-term etiology and whole-body pathogenicity, an important step in the development of new preventive and therapeutic food patterns in humans is to study their potential in preclinical models [45]. Previously, we showed that consumption of pulses exerts both molecular and systemic protection from weight gain in a rodent model for dietary-induced obesity—the C57BL/6J mice—despite an obesogenic dietary environment. Bean Cuisine was designed for humans following our latest reports that 35% of total dietary protein provided by pulses achieves a sufficient dose to observe healthful changes. Gut microbiota emerged as an important mediator of pulse effects, so the next logical step was to conduct confirmatory hypothesis testing that Bean Cuisine formulations can induce a positive shift in the gut microbial community in the same model. To avoid selection, performance, detection, and attribution biases, we replicated our previous studies using Bean Cuisine in lieu of purified diet formulations. To reject our hypothesis that Bean Cuisine menus will not substantially alter the gut microbial community, non-pulse foods included in the control group were comparable to choices of regularly consumed pulse-free diets. The pulse-free control herein, thus, does not represent an “unhealthy” dietary pattern by the composition of the recipe per se. Analysis of the microbiome leads us to conclude that Bean Cuisine offers a two-week meal plan to enhance someone’s diet with beneficial bacteria-inducing pulses without extreme deviation from a regular diet. 

### 4.4. Limitations

The C57BL/6J mouse model was successfully used in pioneering studies that drove initial insights into gut microbial contributions to the development of obesity and related metabolic diseases, and this mouse model remains an often-used tool for initial investigations of the effect of the diet on overall health, mediated by the intestinal microbiota [46]. Despite the 85% shared genetics, the overlap in the general intestinal layout and function, and phylum-level similarity of the bacterial community makeup between mice and humans, distinct sizes, dietary habits, and metabolic rates are the main drivers of translatability issues of the findings from rodent models to human populations. Thus, even though we report individual differences in bacterial genera and their functional predictions, the main focus for interpretation is on the magnitude of change in the gut microbial ecosystem that takes place after the inclusion of pulse in the diet via Bean Cuisine. As such, the findings reported herein allowed us to reject the null hypothesis that the pulses are not potent enough to elicit detectable microbial changes compared to a pulse-free control in a diverse and variable dietary setting oriented for human consumption. 

In contrast to the taxonomic evaluation of microbial communities based on the 16S rRNA gene amplicon data from murine cecal contents, the reconstruction of their functional contributions was purely bioinformatic and predictive in nature. PICRUSt2 uses taxonomic assignments of the observed amplicon data to predict the presence and abundance of functional genes based on publicly available metagenomic information for those taxa. As such, the tool simplifies and automatizes the process of researching every observed taxon individually and assembling the functional capacities shared across multiple bacterial taxa to infer the potential functional net output of microbial community in the experimental group. While there are no confidence values for pathway predictions for the community-wide abundances of genes, PICRUSt2-derived functional reconstruction output significantly correlates with pathways reconstructed from shotgun metagenomic data [22,47], albeit underestimating gene frequencies of some pathways [47]. Given these limitations, we focused primarily on the groups’ most significant differences in functional patterns to reveal the potential metabolic capacities of the Bean Cuisine-induced microbial communities. Future studies using shotgun metagenomics are necessary to assess the functional gene content of observed microbial genomes with a greater degree of accuracy. 

## 5. Conclusions

The introduction of pulses into a dietary pattern, i.e., ensuring regular provision and consistent exposure to pulses and the dietary components and bioactive phytochemicals they contain, has the capacity to improve the stability of the microbial community and the maintenance of eubiosis, which can improve metabolic health and strengthen the protection of the body from dietary insults and the stresses of the daily activities of living. The results of this study performed on female dietary-induced obesity model mice serve as a proof of concept that a mixed pulse food pattern is likely to maximize the diversity of the gut microbiome and that the benefits will involve the metabolism of vitamins, amino acids, short-chain fatty acids, and other mediators of lipid metabolism. This work should be confirmed in male mice. The work reported herein also underscores the potential of the Bean Cuisine food pattern in conferring health benefits for the human population and provides a foundation for investigating this multi-pulse food pattern in community and clinical settings. 

## Figures and Tables

**Table 1:** Proximate analysis (freeze-dried diet).

Diet	% Dry Matter	% Crude Protein	% Crude Fiber	% Fat	% Ash	% Moisture	Nitrogen-Free Extract
CTRL	96.64	25.1	5.4	18.9	4.74	3.36	45.8
D01	95.78	24.3	4.2	9.10	5.03	4.22	57.3
D02	96.07	23.3	5.8	10.4	4.24	3.93	56.2
D03	96.22	19.2	4.7	12.1	3.75	3.78	60.2
D04	95.93	23.4	4.9	15.9	4.11	4.07	51.6
D05	95.44	17.6	4.8	8.50	4.50	4.56	64.5
D06	97.42	20.5	6.4	15.1	4.47	2.58	53.5
D07	95.62	23.5	6.8	13.8	5.13	4.38	50.8
D08	97.16	23.7	6.2	10.1	5.40	2.84	54.6
D09	96.01	20.6	6.7	14.1	4.36	3.99	54.2
D10	96.93	22.3	9.7	12.9	5.08	3.07	50.0
D11	97.49	23.4	5.4	10.7	4.03	2.51	56.5
D12	97.22	19.6	5.9	8.70	4.31	2.78	61.5
D13	95.40	22.2	7.3	10.8	4.47	4.60	55.3
D14	96.98	19.3	7.0	8.60	3.74	3.02	61.4

**Table 2:** Results of the ANCOM-BC analysis of the cofactor and vitamin metabolism pathways based on the prediction of genes with KEGG IDs abundance in the cecal microbiota in mice.

Pathway	(Intercept)	D01	D02	D03	D04	D05	D06	D07	D08	D09	D10	D11	D12	D13	D14
Biotin biosynthesis, BioI pathway, long-chain-acyl-ACP => pimeloyl-ACP => biotin	−0.07	***0.35***	***0.16***	***0.27***	***0.17***	***0.21***	***0.27***		***0.15***	***0.27***	***0.16***	***0.17***		***0.27***	***0.44***
Biotin biosynthesis, BioU pathway, pimeloyl-ACP/CoA => biotin	−0.07	***0.35***	***0.16***	***0.27***	***0.17***	***0.21***	***0.27***		***0.15***	***0.27***	***0.16***	***0.17***		***0.27***	***0.44***
Biotin biosynthesis, BioW pathway, pimelate => pimeloyl-CoA => biotin	0.12	***0.37***	***0.15***	***0.26***	***0.16***	***0.20***	***0.24***			***0.28***		***0.18***		***0.25***	***0.41***
Biotin biosynthesis, pimeloyl-ACP/CoA => biotin	0.12	***0.37***	***0.15***	***0.26***	***0.16***	***0.20***	***0.24***			***0.28***		***0.18***		***0.25***	***0.41***
C1-unit interconversion, eukaryotes	0.21	***−0.07***						***−0.15***		***−0.15***					
C1-unit interconversion, prokaryotes	1.35							***−0.13***							
Cobalamin biosynthesis, aerobic, uroporphyrinogen III => precorrin 2 => cobyrinate a,c-diamide	0.81		***0.31***		***0.28***		***0.19***	***0.14***						***0.22***	***0.33***
Cobalamin biosynthesis, anaerobic, uroporphyrinogen III => sirohydrochlorin => cobyrinate a,c-diamide	1.10		***0.40***		***0.34***		***0.27***	***0.22***	***0.15***				***0.13***	***0.28***	***0.43***
Cobalamin biosynthesis, cobyrinate a,c-diamide => cobalamin	0.67	***0.21***	***0.43***	***0.13***	***0.36***	***0.14***	***0.38***	***0.29***	***0.21***	***0.14***	***0.18***		***0.12***	***0.34***	***0.55***
Coenzyme A biosynthesis, archaea, 2-oxoisovalerate => 4-phosphopantoate => CoA	0.89		***−0.09***					***−0.15***							
Coenzyme A biosynthesis, pantothenate => CoA	1.66							***−0.11***							
Heme biosynthesis, animals and fungi, glycine => heme	−0.25	***0.24***	***0.50***	***0.24***	***0.44***	***0.21***	***0.50***	***0.37***	***0.33***	***0.19***	***0.29***		***0.24***	***0.47***	***0.58***
Heme biosynthesis, bacteria, glutamyl-tRNA => coproporphyrin III => heme	0.65		***0.23***		***0.20***		***0.21***	***0.14***					***0.11***	***0.22***	***0.29***
Heme biosynthesis, plants and bacteria, glutamate => heme	1.59				***0.08***		***0.11***							***0.13***	***0.16***
Lipoic acid biosynthesis, animals and bacteria, octanoyl-ACP => dihydrolipoyl-H => dihydrolipoyl-E2	−2.10	***0.51***		***0.47***		***0.44***	***0.38***		***0.33***	***0.49***	***0.43***	***0.26***		***0.40***	***0.57***
Lipoic acid biosynthesis, eukaryotes, octanoyl-ACP => dihydrolipoyl-H	−2.11	***0.52***		***0.48***		***0.45***	***0.39***		***0.34***	***0.49***	***0.43***	***0.27***		***0.40***	***0.58***
Lipoic acid biosynthesis, octanoyl-CoA => dihydrolipoyl-E2	−2.11	***0.52***		***0.48***		***0.45***	***0.39***		***0.34***	***0.49***	***0.43***	***0.27***		***0.40***	***0.58***
Lipoic acid biosynthesis, plants and bacteria, octanoyl-ACP => dihydrolipoyl-E2/H	−1.41	***0.52***		***0.48***		***0.44***	***0.39***		***0.34***	***0.49***	***0.43***	***0.27***		***0.40***	***0.58***
L-threo-Tetrahydrobiopterin biosynthesis, GTP => L-threo-BH4	−0.46	***0.31***		***0.31***	***0.11***	***0.14***	***0.40***	***0.13***	***0.18***	***0.29***	***0.23***	***0.22***		***0.31***	***0.44***
Menaquinone biosynthesis, chorismate (+ polyprenyl-PP) => menaquinol	0.21	***0.41***	***0.26***	***0.35***	***0.19***	***0.17***	***0.31***	***0.11***	***0.24***	***0.27***	***0.22***	***0.16***	***0.16***	***0.20***	***0.29***
Menaquinone biosynthesis, futalosine pathway	−2.20	***1.12***	***0.74***	***1.47***	***0.55***		***1.32***	***0.84***	***1.24***	***0.97***	***1.28***	***0.65***		***1.13***	***1.48***
Menaquinone biosynthesis, modified futalosine pathway	−2.43	***1.12***	***0.74***	***1.47***	***0.55***		***1.32***	***0.84***	***1.24***	***0.97***	***1.28***	***0.65***		***1.13***	***1.48***
Molybdenum cofactor biosynthesis, GTP => molybdenum cofactor	−0.85		***0.44***	***−0.15***	***0.39***		***0.43***	***0.32***			***0.22***		***0.14***	***0.30***	***0.37***
NAD biosynthesis, aspartate => quinolinate => NAD	1.59		***−0.14***		***−0.09***			***−0.14***							
NAD biosynthesis, tryptophan => quinolinate => NAD	1.12	***0.09***		***0.07***			***0.11***	***−0.07***						***0.06***	***0.07***
Nicotinate degradation, nicotinate => fumarate	−5.39	***1.20***				***−0.80***								***−1.37***	
Pantothenate biosynthesis, 2-oxoisovalerate/spermine => pantothenate	0.80		***−0.18***		***−0.11***		***−0.10***	***−0.16***							
Pantothenate biosynthesis, valine/L-aspartate => pantothenate	1.04	***0.06***	***−0.15***	***0.05***	***−0.07***			***−0.09***		***0.08***				***0.06***	***0.07***
Phylloquinone biosynthesis, chorismate (+ phytyl-PP) => phylloquinol	−0.80	***0.38***		***0.18***		***0.16***				***0.36***		***0.22***			***0.20***
Pimeloyl-ACP biosynthesis, BioC-BioH pathway, malonyl-ACP => pimeloyl-ACP	1.41	***0.08***			***0.06***		***0.12***			***0.05***				***0.10***	***0.18***
Pyridoxal-P biosynthesis, erythrose-4P => pyridoxal-P	0.60	***0.18***	***−0.11***	***0.13***		***0.12***	***0.09***			***0.16***		***0.09***		***0.12***	***0.17***
Pyridoxal-P biosynthesis, R5P + glyceraldehyde-3P + glutamine => pyridoxal-P	−1.64	***0.73***		***0.80***		***0.65***	***0.57***	***0.62***	***0.90***	***0.74***	***0.87***		***0.49***	***0.82***	***0.71***
Riboflavin biosynthesis, fungi, GTP => riboflavin/FMN/FAD	−0.42	***0.36***	***0.32***	***0.24***	***0.30***	***0.29***	***0.33***	***0.21***	***0.25***	***0.23***	***0.23***			***0.36***	***0.53***
Riboflavin biosynthesis, plants and bacteria, GTP => riboflavin/FMN/FAD	0.94	***0.22***	***0.18***	***0.15***	***0.16***	***0.17***	***0.20***		***0.15***	***0.12***	***0.13***		***0.07***	***0.20***	***0.31***
Siroheme biosynthesis, glutamyl-tRNA => siroheme	0.68		***0.21***		***0.18***		***0.18***	***0.11***					***0.11***	***0.17***	***0.27***
Tetrahydrobiopterin biosynthesis, GTP => BH4	−0.46	***0.31***		***0.31***	***0.11***	***0.14***	***0.40***	***0.13***	***0.18***	***0.29***	***0.23***	***0.22***		***0.31***	***0.44***
Tetrahydrofolate biosynthesis, GTP => THF	1.49	***0.16***	***0.07***	***0.18***	***0.07***	***0.08***	***0.18***		***0.13***	***0.13***	***0.13***	***0.09***	***0.05***	***0.16***	***0.23***
Tetrahydrofolate biosynthesis, mediated by PTPS, GTP => THF	0.63	***0.08***	***0.07***	***0.11***	***0.07***		***0.17***		***0.10***		***0.08***			***0.13***	***0.19***
Tetrahydrofolate biosynthesis, mediated by ribA and trpF, GTP => THF	0.57	***0.15***	***0.06***	***0.12***	***0.07***	***0.09***	***0.13***		***0.10***	***0.08***	***0.08***			***0.11***	***0.16***
Thiamine biosynthesis, archaea, AIR (+ NAD+) => TMP/TPP	1.01	***0.08***		***0.05***		***0.08***		***−0.09***							***0.11***
Thiamine biosynthesis, plants, AIR (+ NAD+) => TMP/thiamine/TPP	0.43	***0.12***		***0.10***		***0.07***	***0.09***		***0.13***	***0.09***	***0.10***			***0.11***	***0.11***
Thiamine biosynthesis, prokaryotes, AIR (+ DXP/glycine) => TMP/TPP	1.43							***−0.12***							***0.06***
Thiamine biosynthesis, prokaryotes, AIR (+ DXP/tyrosine) => TMP/TPP	1.54	***0.08***		***0.05***		***0.07***		***−0.08***						***0.08***	***0.11***
Thiamine biosynthesis, pyridoxal-5P => TMP/thiamine/TPP	0.16							***−0.16***							***−0.10***
Thiamine salvage pathway, HMP/HET => TMP	0.97					***0.07***		***−0.08***							
Tocopherol/tocotorienol biosynthesis, homogentisate + phytyl/geranylgeranyl-PP => tocopherol/tocotorienol	−9.34	***0.15***													
Ubiquinone biosynthesis, prokaryotes, chorismate (+ polyprenyl-PP) => ubiquinol	−0.67	***0.52***	***0.51***	***0.57***	***0.31***	***0.31***	***0.61***	***0.38***	***0.43***	***0.30***	***0.44***		***0.29***	***0.40***	***0.62***

Values indicate log_2_ fold change in Bean Cuisine menus vs. pulse-free control. Color signifies the direction of change: orange—increased, and blue—decreased in the respective menu compared to control. Features that were significantly upregulated in multiple diet groups are highlighted in green. Only differences that reached *q*-value < 0.05 are present in the table. Full analysis results are available in the Supplementary Table S6.

**Table 3:** Results of ANCOM-BC analysis of the sulfur metabolism pathways based on PICRUSt2-based prediction of KEGG genes abundance in Bean Cuisine-driven cecal microbiota in mice.

	Pathway	Name	KEGG	D01	D02	D03	D04	D05	D06	D07	D08	D09	D10	D11	D12	D13	D14
**Sulfur metabolism**	**Assimilatory sulfate reduction, sulfate => H_2_S**	cysNC; bifunctional enzyme CysN/CysC [EC:2.7.7.4 2.7.1.25]	K00955	***1.21***	***3.16***	***1.42***	***1.00***	***1.79***	***0.90***					***1.69***			
		cysC; adenylylsulfate kinase [EC:2.7.1.25]	K00860		***−0.95***		***−0.64***					***0.33***					***0.37***
		cysD; sulfate adenylyltransferase subunit 2 [EC:2.7.7.4]	K00957		***−0.57***		***−0.70***	***0.29***				***0.40***					***0.42***
		cysH; phosphoadenosine phosphosulfate reductase [EC:1.8.4.8 1.8.4.10]	K00390	***0.47***								***0.55***					***0.42***
		cysI; sulfite reductase (NADPH) hemoprotein beta-component [EC:1.8.1.2]	K00381	***0.56***								***1.28***					
		cysJ; sulfite reductase (NADPH) flavoprotein alpha-component [EC:1.8.1.2]	K00380	***0.56***								***1.28***					
		cysN; sulfate adenylyltransferase subunit 1 [EC:2.7.7.4]	K00956		***−1.06***		***−0.76***			***−0.37***		***0.32***			***−0.40***		
		sat, met3; sulfate adenylyltransferase [EC:2.7.7.4]	K00958				***−0.27***										
		sir; sulfite reductase (ferredoxin) [EC:1.8.7.1]	K00392				***−0.27***										
	**Dissimilatory sulfate reduction, sulfate => H_2_S**	aprA; adenylylsulfate reductase, subunit A [EC:1.8.99.2]	K00394	***1.21***		***1.43***				***1.05***			***1.12***				***1.54***
		aprB; adenylylsulfate reductase, subunit B [EC:1.8.99.2]	K00395	***1.21***		***1.43***				***1.05***			***1.12***				***1.54***
		sat, met3; sulfate adenylyltransferase [EC:2.7.7.4]	K00958				***−0.27***										
**Metabolic capacity**	**Sulfate-sulfur assimilation**	cysA; sulfate transport system ATP-binding protein [EC:3.6.3.25]	K02045		***0.63***		***−0.33***		***0.61***	***0.92***					***0.44***		***0.77***
		cysP, sbp; sulfate transport system substrate-binding protein	K02048	***1.32***	***1.58***	***1.56***		***1.22***		***1.07***			***1.12***				***1.59***
		cysU; sulfate transport system permease protein	K02046	***1.32***	***1.58***	***1.56***		***1.22***		***1.07***			***1.12***				***1.59***
		cysW; sulfate transport system permease protein	K02047	***1.32***	***1.58***	***1.56***		***1.22***		***1.07***			***1.12***				***1.59***

Values indicate log_2_ fold change in Bean Cuisine menus vs. pulse-free control. Color signifies the direction of change: orange—increased, and blue—decreased in respective menu compared to control. Only differences that reached *q*-value < 0.05 are present in the table. Full analysis results are available in the Supplementary Table S5.

**Table 4:** Results of ANCOM-BC analysis of the amino acid metabolism pathways based on PICRUSt2-based prediction of KEGG genes abundance in Bean Cuisine-driven cecal microbiota in mice.

	Pathway	D01	D02	D03	D04	D05	D06	D07	D08	D09	D10	D11	D12	D13	D14
**Arginine and proline metabolism**	Proline degradation, proline => glutamate							***0.36***						***0.45***	
	Creatine pathway			***−0.69***	***−0.04***						***0.24***	***0.03***			
	Arginine succinyltransferase pathway, arginine => glutamate													***−0.10***	
	Proline biosynthesis, glutamate => proline		***−0.22***			***0.00***									***−0.07***
	Arginine biosynthesis, glutamate => acetylcitrulline => arginine		***−0.18***			***0.00***									***−0.05***
	Ornithine biosynthesis, glutamate => ornithine		***−0.19***			***0.02***									***−0.03***
	Proline metabolism		***−0.19***			***0.02***									***−0.02***
	Arginine biosynthesis, ornithine => arginine					***0.01***									***−0.05***
	Urea cycle					***0.01***									***−0.04***
**Aromatic amino acid metabolism**	Tryptophan biosynthesis, chorismate => tryptophan	***0.34***	***0.27***	***0.31***	***0.28***	***0.18***	***0.33***	***0.23***	***0.28***	***0.29***	***0.23***	***0.18***	***0.17***	***0.35***	
	Tryptophan metabolism, tryptophan => kynurenine => 2-aminomuconate										***0.11***	***0.38***	***−0.45***		
	Homoprotocatechuate degradation, homoprotocatechuate => 2-oxohept-3-enedioate													***−0.10***	
	Tyrosine degradation, tyrosine => homogentisate	***−0.68***	***−0.96***		***−0.55***	***−0.22***	***−0.95***	***−0.75***	***−0.57***		***−0.45***	***−0.21***	***−0.24***		***−1.24***
	Phenylalanine biosynthesis, chorismate => phenylpyruvate => phenylalanine		***−0.30***		***−0.16***	***−0.02***	***−0.20***						***−0.06***		***−0.16***
	Tyrosine biosynthesis, chorismate => arogenate => tyrosine	***−0.27***	***−0.42***		***−0.21***	***−0.06***							***−0.06***		***−0.34***
	Tyrosine biosynthesis, chorismate => HPP => tyrosine		***−0.29***		***−0.15***	***−0.03***							***−0.05***		***−0.17***
	Shikimate pathway, phosphoenolpyruvate + erythrose-4P => chorismate					***0.01***									***0.01***
**Branched-chain amino acid metabolism**	Leucine degradation, leucine => acetoacetate + acetyl-CoA	***0.15***	***−0.13***	***0.10***	***−0.05***		***0.08***	***0.01***	***0.09***	***0.17***		***0.08***	***0.03***		
	Isoleucine biosynthesis, pyruvate => 2-oxobutanoate		***−0.18***			***0.03***									***0.00***
	Leucine biosynthesis, 2-oxoisovalerate => 2-oxoisocaproate		***−0.19***			***0.02***									***−0.03***
	Isoleucine biosynthesis, threonine => 2-oxobutanoate => isoleucine					***0.01***									***0.03***
	Valine/isoleucine biosynthesis, pyruvate => valine / 2-oxobutanoate => isoleucine					***0.01***									***0.05***
**Cysteine and methionine metabolism**	Cysteine biosynthesis, homocysteine + serine => cysteine	***0.80***			***0.26***	***0.49***	***0.58***	***0.77***	***0.92***	***0.87***	***0.90***	***0.54***	***0.72***	***0.74***	
	Methionine degradation	***0.10***	***0.14***		***0.12***	***0.02***	***0.20***	***0.06***	***0.11***		***0.11***	***0.07***	***0.07***	***0.14***	
	Cysteine biosynthesis, serine => cysteine	***−0.14***	***−0.31***		***−0.17***	***−0.03***	***−0.18***		***−0.15***		***−0.12***	***−0.04***	***−0.11***		***−0.13***
	Methionine salvage pathway					***−0.03***							***−0.01***		***−0.09***
	Methionine biosynthesis, aspartate => homoserine => methionine					***0.02***									***−0.01***
	Cysteine biosynthesis, methionine => cysteine					***−0.03***									***−0.07***
	Ethylene biosynthesis, methionine => ethylene					***−0.01***									***0.00***
**Histidine metabolism**	Histidine degradation, histidine => N-formiminoglutamate => glutamate	***0.58***		***0.37***	***−0.03***		***0.32***	***0.04***	***0.33***				***−0.06***	***0.27***	
	Histidine biosynthesis, PRPP => histidine	***−0.13***	***−0.31***		***−0.17***	***−0.05***	***−0.18***		***−0.14***				***−0.08***		***−0.18***
**Lysine metabolism**	Lysine degradation, bacteria, L-lysine => succinate	***0.21***	***0.55***		***0.39***	***0.14***	***0.52***	***0.27***	***0.56***		***0.46***	***0.07***	***0.37***	***0.27***	
	Lysine biosynthesis, AAA pathway, 2-oxoglutarate => 2-aminoadipate => lysine		***0.31***	***0.14***	***0.31***	***0.28***		***0.20***				***0.02***	***0.10***	***0.29***	
	Lysine biosynthesis, DAP dehydrogenase pathway, aspartate => lysine														
	Lysine degradation, bacteria, L-lysine => glutarate => succinate/acetyl-CoA	***−0.23***	***−0.15***	***−0.26***	***−0.15***	***−0.19***	***−0.20***	***−0.39***	***−0.21***			***−0.15***	***−0.07***		***−0.41***
	Lysine biosynthesis, succinyl-DAP pathway, aspartate => lysine		***−0.21***			***0.00***									***−0.08***
	Lysine biosynthesis, acetyl-DAP pathway, aspartate => lysine		***−0.18***			***0.00***									***−0.05***
	Lysine biosynthesis, DAP aminotransferase pathway, aspartate => lysine		***−0.17***			***0.02***									***0.00***
**Other amino acid metabolism**	GABA (gamma-Aminobutyrate) shunt	***0.42***	***0.49***	***0.39***	***0.31***	***0.16***	***0.48***		***0.34***		***0.27***	***0.10***	***0.22***	***0.21***	
	Glutathione biosynthesis, glutamate => glutathione	***0.86***		***0.97***		***0.57***	***0.50***	***0.56***	***1.01***		***0.98***	***0.44***	***0.62***	***0.76***	
	Hydroxyproline degradation, trans-4-hydroxy-L-proline => 2-oxoglutarate													***−0.10***	
**Polyamine biosynthesis**	Polyamine biosynthesis, arginine => agmatine => putrescine => spermidine	***0.45***	***0.40***	***0.36***	***0.33***	***0.41***	***0.43***	***0.36***	***0.42***			***0.06***	***0.15***	***0.52***	
	Polyamine biosynthesis, arginine => ornithine => putrescine		***0.73***		***0.47***		***0.51***	***0.13***					***0.27***		
	GABA biosynthesis, eukaryotes, putrescine => GABA		***−0.14***			***0.05***									
	GABA biosynthesis, prokaryotes, putrescine => GABA													***−0.10***	
**Serine and threonine metabolism**	Glycine cleavage system	***0.41***	***0.13***	***0.32***	***0.12***	***0.24***	***0.40***	***0.28***	***0.24***	***0.38***	***0.25***	***0.23***	***0.18***	***0.35***	
	Betaine biosynthesis, choline => betaine		***0.54***		***1.72***	***1.65***			***0.24***		***1.68***	***0.34***		***3.04***	
	Serine biosynthesis, glycerate-3P => serine	***0.01***	***−0.22***			***0.00***									***−0.07***
	Ectoine biosynthesis, aspartate => ectoine		***−0.28***		***−0.15***	***0.02***							***−0.05***		***−0.09***
	Threonine biosynthesis, aspartate => homoserine => threonine		***−0.28***		***−0.14***	***0.00***							***−0.05***		***−0.11***
	Betaine degradation, bacteria, betaine => pyruvate					***−0.01***									***0.00***

Values indicate log_2_ fold change in Bean Cuisine menus vs. pulse-free control. Color signifies the direction of change: orange—increased, and blue—decreased in respective menu compared to control. Features that were significantly upregulated in multiple diet groups are highlighted in green, and those suppressed—in orange. Only differences that reached *q*-value < 0.05 are present in the table. Full analysis results are available in the Supplementary Table S6.

## Data Availability

The original contributions presented in the study are included in the article, further inquiries can be directed to the corresponding author.

## Supplementary Materials

The following supporting information can be downloaded at: https://www.mdpi.com/article/10.3390/nu16183178/s1, Supplementary Materials S1: Original Bean Cuisine—Nutrient Analysis; Supplementary Materials S2: Bean Cuisine—Meals Report; Supplementary Materials S3: Pulse-free control—Nutrient Analysis; Supplementary Table S1: Results of the statistical analyses of the β-diversity results between pulse-free control and pulse-containing cohort of equivalent menus; Supplementary Table S2: Differential analysis of cecal microbiota (at the Genus level) using LEfSe (non-strict mode); Supplementary Table S3: Differential analysis of cecal microbiota (at the Genus level) in each of the fourteen pulse-containing menus vs. control (ANCOM-BC); Supplementary Table S4: Differential analysis of PICRUSt2-pathways (EC) in each of the fourteen pulse-containing menus vs. control (ANCOM-BC); Supplementary Table S5: Differential analysis of PICRUSt2-KO genes in each of the fourteen pulse-containing menus vs. control (ANCOM-BC); Supplementary Table S6: Differential analysis of PICRUSt2-pathways (KEGG) in each of the fourteen pulse-containing menus vs. control (ANCOM-BC); Supplementary Table S7: Correlation analyses between cecal microbiota, PICRUSt2-pathways, and foods from the Bean Cuisine menus vs pulse-free control using Spearman’s method; Supplementary Table S8: Information on the outlier/excluded animals; Supplementary Table S9: Power analysis.
