# Supplementary material for: Impact of a Pulse-Enriched Human Cuisine on Functional Attributes of the Gut Microbiome Using a Preclinical Model of Dietary-Induced Chronic Diseases
Source: Nutrients. 2024 Sep 20;16(18):3178. doi: 10.3390/nu16183178 (PMC11434987; doi:10.3390/nu16183178)
Supplement: Supplementary file 1 [file nutrients-16-03178-s001.zip › Supplementary Materials.pdf]

Menu Template Nutrient Analysis

Total Days: 14
Total Foods: 151
Avg. Daily Kcals: 1999.343
Name: Original Bean Cuisine

Nutrient	Value	Unit	Goal	%
Weight	2143.808	g		
Kilocalories	1999.343	kcal	2000.000	100 %
Kilojoules	8262.630	kj		
Protein	95.639	g	50.000	191 %
Carbohydrate	291.866	g	275.000	106 %
Available Carbohydrate	0.017	g		
Fat, Total	58.682	g	78.000	75 %
Alcohol	0.249	g		
Cholesterol	209.187	mg	300.000	70 %
Saturated Fat	14.324	g	20.000	72 %
Monounsaturated Fat	17.924	g		
Polyunsaturated Fat	13.644	g		
SFA 4:0	0.225	g		
SFA 6:0	0.151	g		
SFA 8:0	0.094	g		
SFA 10:0	0.219	g		
SFA 12:0, Lauric	0.238	g		
SFA 14:0	0.767	g		
SFA 15:0 Pentadecanoic acid	0.039	g		
SFA 24:0 Lignoceric Acid	0.003	g		
SFA 16:0, Palmitic	6.742	g		
SFA 17:0	0.052	g		
SFA 18:0, Stearic	2.286	g		
SFA 20:0	0.041	g		
SFA 22:0	0.014	g		
MFA 14:1	0.032	g		
MFA 16:1	0.481	g		
MFA 18:1, Oleic	16.754	g		
MFA 20:1	0.160	g		
MFA 22:1	0.010	g		
PFA 18:2, Linoleic	11.382	g		
PFA 18:3, Linolenic	1.799	g		
PFA 18:4	0.004	g		
PFA 20:4	0.104	g		
PFA 20:5, EPA	0.010	g		
PFA 22:5	0.008	g		
PFA 22:6, DHA	0.041	g		
Trans Fatty Acid	0.328	g		
Sum of Trans Fat and Sat Fat	9.876	g		
Omega 3 Polyunsat Fat, Tot	0.000	g		
Omega 6 Polyunsat Fat, Tot	0.000	g		
Sodium	1933.959	mg	2300.000	84 %
Potassium	5142.688	mg	4700.000	109 %
Salt	4.838	g		
Chloride			2300.000	
Vitamin A (RE)	1658.216	RE		

Nutrient	Value	Unit	Goal	%
Cobalamin (Vitamin B12)	6.261	mcg	2.400	261 %
Biotin	21.298	mcg	30.000	71 %
Pantothenic Acid	5.505	mg	5.000	110 %
Vitamin K	267.241	mcg	120.000	223 %
Phosphorus	1524.381	mg	1250.000	122 %
Iodine		mcg	150.000	
Magnesium	498.766	mg	420.000	119 %
Zinc	10.911	mg	11.000	99 %
Copper	1.978	mg	0.900	220 %
Manganese	4.759	mg	2.300	207 %
Selenium	81.934	mcg	55.000	149 %
Fluoride	75.690	mcg		
Chromium	0.048	mg	0.035	138 %
Molybdenum	40.987	mcg	45.000	91 %
Choline	372.470	mg	550.000	68 %
Chlorine		mg		
Sulfur		mg		
Cobalt		mcg		
Boron		mcg		
Nickel		mcg		
Dietary Fiber, Total	61.469	g	28.000	220 %
Soluble Fiber	2.762	g		
Insoluble Fiber	4.571	g		
Crude Fiber	19.461	g		
Sugar, Total	88.943	g		
Added Sugars	11.893	g	50.000	24 %
Glucose	19.790	g		
Galactose	1.262	g		
Fructose	21.934	g		
Sucrose	20.247	g		
Lactose	7.737	g		
Maltose	0.863	g		
Sugar Alcohol	0.000	g		
Other Carbohydrate	0.000	g		
Sorbitol	0.000	g		
Xylitol	0.000	g		
Inositol		mg		
Tryptophan	848.866	mg		
Threonine	2911.142	mg		
Isoleucine	3269.393	mg		
Leucine	5749.628	mg		
Lysine	5077.066	mg		
Methionine	1354.678	mg		
Cystine	869.929	mg		
Phenylalanine	3636.166	mg		
Tyrosine	2320.688	mg		
Valine	3931.118	mg		
Arginine	4561.384	mg		

Nutrient Goal Template
DAILY VALUES/RDI - ADULT/CHILD

Analyzed by
Original Bean Cuisine

Exchanges

Bread/Starch	2.00
Fat	0.50
Fruit	3.00
Meat-High Fat	0.00
Meat-Medium Fat	0.00
Meat-Very Lean	0.00
Milk-Skim	0.50
Other Carbohydrate	0.00
Vegetables	1.00

Menu Template Nutrient Analysis

Total Days: 14
Total Foods: 151
Avg. Daily Kcals: 1999.343
Name: Original Bean Cuisine

Nutrient	Value	Unit	Goal	%
Vitamin A (IU)	14800.530	IU	5000.000	296 %
Vitamin A (RAE)	1866.178	mcg	900.000	207 %
Total Carotenoid	0.000	RE		
Beta-Carotene	8930.838	mcg		
Alpha-Carotene	1732.957	mcg		
Lutein (+ Zeaxanthin)	4636.036	mcg		
Beta-Cryptoxanthin	187.945	mcg		
Lycopene	7268.183	mcg		
Vitamin C	193.251	mg	90.000	215 %
Calcium	1450.116	mg	1300.000	112 %
Iron	20.203	mg	18.000	112 %
Vitamin D (ug)	7.556	mcg	20.000	38 %
Vitamin D (IU)	304.746	IU	400.000	76 %
Vitamin E (mg)	0.269	mg	20.000	1 %
Vitamin E (IU)	0.401	IU	30.000	1 %
Vitamin E (Alpha-Tocopherol)	10.357	mg	15.000	69 %
Beta Tocopherol	0.098	mg		
Gamma Tocopherol	2.232	mg		
Delta Tocopherol	0.218	mg		
Alpha Tocotrienol	0.212	mg		
Beta Tocotrienol	0.289	mg		
Gamma Tocotrienol	0.257	mg		
Delta Tocotrienol	0.006	mg		
Thiamin	3.083	mg	1.200	257 %
Riboflavin	3.273	mg	1.300	252 %
Niacin	14.902	mg	20.000	75 %
Niacin Equivalent	32.267	mg	16.000	202 %
Pyridoxine (Vitamin B6)	2.867	mg	1.700	169 %
Folate (Total)	805.451	mcg	400.000	201 %
Folate (DFE)	1010.164	mcg	400.000	253 %
Folic Acid	133.862	mcg		
Food Folate	786.102	mcg		

Nutrient	Value	Unit	Goal	%
Histidine	1994.016	mg		
Alanine	3392.822	mg		
Aspartic Acid	7924.574	mg		
Glutamic Acid	12651.570	mg		
Glycine	2884.937	mg		
Proline	4020.810	mg		
Serine	3746.584	mg		
Glutamine	0.000	mg		
Taurine	0.000	mg		
Hydroxyproline	24.793	mg		
Cysteine	0.000	mg		
Moisture	1427.764	g		
Ash	16.082	g		
Caffeine	3.401	mg		
Osmolality		mo		
Theobromine	30.414	mg		
Betaine	23.225	mg		
Phytosterols	48.141	mg		
Stigmasterol	1.428	mg		
Campesterol	3.595	mg		
Beta-sitosterol	22.840	mg		
Epigallocatechin-3-gallate		mg		
Daidzein	0.015	mg		
Genistein	0.028	mg		
Glycitein	0.000	mg		
Total isoflavones	0.050	mg		
Biochanin A	0.089	mg		
Formononetin	0.014	mg		
Coumestrol	0.001	mg		

Supplementary Materials S2

Menu Template Meals Report

Total Days: 14
Total Foods: 151

Avg. Daily Kcals: 1999.343
Name: Original Bean Cuisine

	Breakfast	Lunch	Dinner	Morning Snack	Afternoon Snack	Evening Snack
Day 1	Chickpea Flour Scramble	Multi-Bean Salad	Zesty Salsa Chicken Pulse Pasta		Olive Bean Dip	
	Avocado, California	Orange	Garden Salad		WHEAT THINS Crackers, Hint of Salt	
	Yogurt, Plain, Low Fat (12 grams protein per 8 ounces)	PLANET OAT Milk, Oatmilk, Original			SILK Plus Omega-3 DHA, soymilk	
	Banana				DOVE PROMISES Candy Bar, Dark Chocolate, Silky Smooth	
	Milk, Low Fat, 1%					
Day 2	Chocolate Black Bean Smoothie	Bean and Tuna Salad Sandwich	Bean and Quinoa Harvest Salad		Crispy Chickpeas	
	Eggs, Hard Boiled	Carrots, Baby	Blueberries		Almonds, Dry Roasted, without Salt Added	
		SILK Plus Omega-3 DHA, soymilk			PLANET OAT Milk, Oatmilk, Original	
					Dates, Medjool	

Menu Template Meals Report

Total Days: 14
Total Foods: 151

Avg. Daily Kcals: 1999.343
Name: Pulse Cuisine_v1_ORIGINAL

	Breakfast	Lunch	Dinner	Morning Snack	Afternoon Snack	Evening Snack
**Day 3**	Apple Cinnamon Lentil Porridge	Bean and Cheese Burrito	Ginger Chicken and Beans		Black Bean Brownie	
	Yogurt, Plain, Low Fat (12 grams protein per 8 ounces)	Watermelon	Rice, Brown, Medium Grain, Cooked		PLANET OAT Milk, Oatmilk, Original	
	Strawberries (Strawberry)	Juice, Lime	Grapes, Red or Green			
**Day 4**	Carrot Cake White Bean Oatmeal	White Bean Waldorf Salad	Bean and Sweet Potato Bake		Savory Chickpea Bread	
	PLANET OAT Milk, Oatmilk, Original	Garden Salad with Chicken	Eggs, Fried		Yogurt, Plain, Low Fat (12 grams protein per 8 ounces)	
		Orange			Blueberries	
**Day 5**	Easy Baked Beans on Toast	Rocky Mountain "Caviar"	Lentil Curry		Buffalo Chickpea Dip	
	Orange	FOOD SHOULD TASTE GOOD Chips, Tortilla, Multigrain	Broccoli		Celery, Stalk	
	Milk, Low Fat, 1%	Apple, Medium	Strawberries (Strawberry)		Carrots	

Menu Template Meals Report

Total Days: 14
Total Foods: 151
Avg. Daily Kcals: 1999.343
Name: Pulse Cuisine_v1_ORIGINAL

	Breakfast	Lunch	Dinner	Morning Snack	Afternoon Snack	Evening Snack
			SILK Plus Omega-3 DHA, soymilk		Dates, Medjool	
**Day 6**	Chickpea Flour Everything Bagel Muffins<br>Grapefruit, Pink or Red<br>SILK Plus Omega-3 DHA, soymilk	Black Bean Salad<br>Eggs, Hard Boiled<br>Kiwi Fruit, Green or Chinese Gooseberries	Bean and Turkey Quesadilla<br>Salsa		Peanut Butter White Bean Blondie<br>PLANET OAT Milk, Oatmilk, Original	
**Day 7**	Pinto Bean Shakshuka<br>Bread, Pita, Whole Wheat<br>Yogurt, Plain, Low Fat (12 grams protein per 8 ounces)	Herby Bean Toast<br>Garden Salad<br>PLANET OAT Milk, Oatmilk, Original	White Bean Chicken Chili<br>Orange		Artichoke Bean Dip<br>Pepper, Bell or Sweet, Red<br>DOVE PROMISES Candy Bar, Dark Chocolate, Silky Smooth<br>Cucumber	
**Day 8**	Strawberry Banana Bean Smoothie	Bean and Rice Salad	Lentil, Broccoli, and Shrimp Bake		Pinto Pumpkin Dip	

Menu Template Meals Report

Total Days: 14
Total Foods: 151
Avg. Daily Kcals: 1999.343
Name: Pulse Cuisine_v1_ORIGINAL

	Breakfast	Lunch	Dinner	Morning Snack	Afternoon Snack	Evening Snack
	Peanuts, dry roasted, without salt	SILK Plus Omega-3 DHA, soymilk<br>Dates, Medjool	Potatoes, Sweet, Baked in Skin<br>Cottage Cheese, Low Fat 1%<br>Chives, Chopped		Pepper, Bell or Sweet, Green<br>PLANET OAT Milk, Oatmilk, Original	
Day 9	Chickpea Dutch Baby<br>PLANET OAT Milk, Oatmilk, Original	Lentil Tacos<br>Yogurt, Plain, Low Fat (12 grams protein per 8 ounces)<br>Mango	Chicken and Bean Cassoulet<br>Blueberries		Snickerdoodle Hummus<br>Apple, Medium	
Day 10	Black Bean Breakfast Burrito<br>SILK Plus Omega-3 DHA, soymilk	Mediterranean Chickpea Salad<br>Carrots, Baby<br>PLANET OAT Milk, Oatmilk, Original	Pinto and Ham Soup<br>Bread, 100% Whole Wheat		Chocolate Cherry Mayocoba Bean Bars<br>Grapefruit, Pink or Red	

Menu Template Meals Report

Total Days: 14
Total Foods: 151
Avg. Daily Kcals: 1999.343
Name: Pulse Cuisine_v1_ORIGINAL

	Breakfast	Lunch	Dinner	Morning Snack	Afternoon Snack	Evening Snack
Day 11	Savory Chickpea Waffle	Super Simple Pinto Bean Burger	Chicken and Pasta with Beans		Split Pea Hummus	
	Banana	Orange	SILK Plus Omega-3 DHA, soymilk		Cauliflower, Chopped	
	PLANET OAT Milk, Oatmilk, Original				Dates, Medjool	
Day 12	Blueberry Banana White Bean Oats	Beany Salad Wrap	Eggy Lentil Pasta		Mayocoba Cookie Dough Dip	
	PLANET OAT Milk, Oatmilk, Original	Watermelon	Grapes, Red or Green		Crackers, Graham, Plain	
		SILK Plus Omega-3 DHA, soymilk				
Day 13	Chickpea Flour Frittata	Mayocoba Stuffed Sweet Potatoes with Tahini Yogurt Sauce	Turkey and Bean Skillet		Lentil Bruschetta with Tomatoes, Basil, and Feta	
	Grapefruit, Pink or Red	Grapes, Red or Green	Juice, Lime			
	PLANET OAT Milk, Oatmilk, Original	SILK Plus Omega-3 DHA, soymilk				

Menu Template Meals Report

Total Days: 14
Total Foods: 151
Avg. Daily Kcals: 1999.343
Name: Pulse Cuisine_v1_ORIGINAL

	Breakfast	Lunch	Dinner	Morning Snack	Afternoon Snack	Evening Snack
**Day 14**	Beany Breakfast Hash<br>Banana<br>SILK Plus Omega-3 DHA, soymilk	Bean and Rice Soup<br>Bread, 100% Whole Wheat	Summery Split Pea and Tomato Salad<br>Watermelon		Black Bean Chocolate Mousse<br>Strawberries (Strawberry)<br>Crackers, Graham, Plain<br>Milk, Low Fat, 1%	

Supplementary Materials S3

Menu Template Nutrient Analysis

Total Days: 4
Total Foods: 43
Avg. Daily Kcals: 2011.525
Name: Pulse_Cuisine_CONTROL_D1-10-13-14

Nutrient	Value	Unit	Goal	%
Weight	2226.576	g		
Kilocalories	2011.525	kcal	2000.000	101 %
Kilojoules	8417.059	kj		
Protein	100.977	g	50.000	202 %
Carbohydrate	217.397	g	275.000	79 %
Available Carbohydrate	0.000	g		
Fat, Total	88.560	g	78.000	114 %
Alcohol	0.235	g		
Cholesterol	628.675	mg	300.000	210 %
Saturated Fat	29.732	g	20.000	149 %
Monounsaturated Fat	26.214	g		
Polyunsaturated Fat	14.023	g		
SFA 4:0	0.671	g		
SFA 6:0	0.502	g		
SFA 8:0	0.341	g		
SFA 10:0	0.839	g		
SFA 12:0, Lauric	0.823	g		
SFA 14:0	2.689	g		
SFA 15:0 Pentadecanoic acid	0.194	g		
SFA 24:0 Lignoceric Acid	0.013	g		
SFA 16:0, Palmitic	13.556	g		
SFA 17:0	0.215	g		
SFA 18:0, Stearic	5.422	g		
SFA 20:0	0.085	g		
SFA 22:0	0.035	g		
MFA 14:1	0.194	g		
MFA 16:1	1.104	g		
MFA 18:1, Oleic	24.187	g		
MFA 20:1	0.177	g		
MFA 22:1	0.003	g		
PFA 18:2, Linoleic	12.204	g		
PFA 18:3, Linolenic	1.462	g		
PFA 18:4	0.000	g		
PFA 20:4	0.234	g		
PFA 20:5, EPA	0.036	g		
PFA 22:5	0.026	g		
PFA 22:6, DHA	0.071	g		
Trans Fatty Acid	0.843	g		
Sum of Trans Fat and Sat Fat	20.113	g		
Omega 3 Polyunsat Fat, Tot	0.000	g		
Omega 6 Polyunsat Fat, Tot	0.000	g		
Sodium	3087.978	mg	2300.000	134 %
Potassium	4350.730	mg	4700.000	93 %
Salt	7.767	g		
Chloride			2300.000	
Vitamin A (RE)	2785.558	RE		

Nutrient	Value	Unit	Goal	%
Cobalamin (Vitamin B12)	8.436	mcg	2.400	352 %
Biotin	17.512	mcg	30.000	58 %
Pantothenic Acid	6.674	mg	5.000	133 %
Vitamin K	325.093	mcg	120.000	271 %
Phosphorus	1468.401	mg	1250.000	117 %
Iodine	5.569	mcg	150.000	4 %
Magnesium	386.981	mg	420.000	92 %
Zinc	11.995	mg	11.000	109 %
Copper	1.400	mg	0.900	156 %
Manganese	2.780	mg	2.300	121 %
Selenium	140.752	mcg	55.000	256 %
Fluoride	106.895	mcg		
Chromium	0.048	mg	0.035	136 %
Molybdenum	20.689	mcg	45.000	46 %
Choline	436.020	mg	550.000	79 %
Chlorine		mg		
Sulfur		mg		
Cobalt		mcg		
Boron		mcg		
Nickel		mcg		
Dietary Fiber, Total	30.107	g	28.000	108 %
Soluble Fiber	3.335	g		
Insoluble Fiber	4.596	g		
Crude Fiber	9.895	g		
Sugar, Total	88.745	g		
Added Sugars	11.775	g	50.000	24 %
Glucose	17.426	g		
Galactose	1.101	g		
Fructose	18.314	g		
Sucrose	21.749	g		
Lactose	11.597	g		
Maltose	0.441	g		
Sugar Alcohol	0.000	g		
Other Carbohydrate	0.000	g		
Sorbitol		g		
Xylitol		g		
Inositol		mg		
Tryptophan	830.779	mg		
Threonine	2689.203	mg		
Isoleucine	2917.614	mg		
Leucine	5181.000	mg		
Lysine	4885.986	mg		
Methionine	1537.711	mg		
Cystine	644.058	mg		
Phenylalanine	2806.694	mg		
Tyrosine	2413.355	mg		
Valine	3448.530	mg		
Arginine	3738.124	mg		

Nutrient Goal Template

DAILY VALUES/RDI - ADULT/CHILD

Analyzed by

Pulse_Cuisine_CONTROL_D1-10-13-14

Exchanges

Bread/Starch	2.00
Fat	1.00
Fruit	3.00
Milk-Skim	1.00
Other Carbohydrate	0.00
Vegetables	0.00

Menu Template Nutrient Analysis

Total Days: 4
Total Foods: 43
Avg. Daily Kcals: 2011.525
Name: Pulse_Cuisine_CONTROL_D1-10-13-14

Nutrient	Value	Unit	Goal	%
Vitamin A (IU)	24074.960	IU	5000.000	481 %
Vitamin A (RAE)	2807.112	mcg	900.000	312 %
Total Carotenoid	0.000	RE		
Beta-Carotene	12741.120	mcg		
Alpha-Carotene	1520.844	mcg		
Lutein (+ Zeaxanthin)	7014.192	mcg		
Beta-Cryptoxanthin	236.442	mcg		
Lycopene	8436.642	mcg		
Vitamin C	191.697	mg	90.000	213 %
Calcium	1688.132	mg	1300.000	130 %
Iron	13.117	mg	18.000	73 %
Vitamin D (ug)	12.981	mcg	20.000	65 %
Vitamin D (IU)	521.859	IU	400.000	130 %
Vitamin E (mg)	9.259	mg	20.000	46 %
Vitamin E (IU)	13.803	IU	30.000	46 %
Vitamin E (Alpha-Tocopherol)	14.449	mg	15.000	96 %
Beta Tocopherol	0.165	mg		
Gamma Tocopherol	3.505	mg		
Delta Tocopherol	0.793	mg		
Alpha Tocotrienol	0.313	mg		
Beta Tocotrienol	0.598	mg		
Gamma Tocotrienol	0.335	mg		
Delta Tocotrienol	0.007	mg		
Thiamin	1.073	mg	1.200	89 %
Riboflavin	2.857	mg	1.300	220 %
Niacin	18.981	mg	20.000	95 %
Niacin Equivalent	30.081	mg	16.000	188 %
Pyridoxine (Vitamin B6)	2.520	mg	1.700	148 %
Folate (Total)	433.094	mcg	400.000	108 %
Folate (DFE)	410.490	mcg	400.000	103 %
Folic Acid	2.520	mcg		
Food Folate	349.867	mcg		

Nutrient	Value	Unit	Goal	%
Histidine	1842.690	mg		
Alanine	3172.086	mg		
Aspartic Acid	6115.220	mg		
Glutamic Acid	11788.870	mg		
Glycine	2490.283	mg		
Proline	4104.103	mg		
Serine	2847.789	mg		
Glutamine	0.000	mg		
Taurine	0.000	mg		
Hydroxyproline	190.365	mg		
Cysteine	0.000	mg		
Moisture	1564.949	g		
Ash	15.570	g		
Caffeine	6.978	mg		
Osmolality		mo		
Theobromine	61.561	mg		
Betaine	51.216	mg		
Phytosterols	50.453	mg		
Stigmasterol	2.916	mg		
Campesterol	3.368	mg		
Beta-sitosterol	15.509	mg		
Epigallocatechin-3-gallate		mg		
Daidzein	0.001	mg		
Genistein	0.006	mg		
Glycitein	0.000	mg		
Total isoflavones	0.008	mg		
Biochanin A	0.001	mg		
Formononetin	0.001	mg		
Coumestrol	0.001	mg		
